# VEGF-D-induced intraosseous lymphangiogenesis drives site-specific heterotopic bone resorption

**DOI:** 10.1073/pnas.2524022123

**Published:** 2026-05-05

**Authors:** Neda Vishlaghi, Danielle Griswold-Wheeler, Sneha Korlakunta, Angelica Vallejo, Monisha Mittal, Yuxiao Sun, Peng Zhao, Yunzhi Peter Yang, Joseph M. Rutkowski, Benjamin Levi, Michael Dellinger

**Affiliations:** ^a^Department of Surgery, UT Southwestern Medical Center, Dallas, TX 75390; ^b^Hamon Center for Therapeutic Oncology Research, UT Southwestern Medical Center, Dallas, TX 75390; ^c^ Department of Orthopedic Surgery, Stanford University, San Francisco, CA 94305; ^d^ Department of Materials Science and Engineering, Stanford University, San Francisco, CA 94305; ^e^ Department of Bioengineering, Stanford University, San Francisco, CA 94305; ^f^ Department of Medical Physiology, Texas A&M University College of Medicine, Bryan, TX 77843

**Keywords:** heterotopic ossification, lymphangiogenesis, VEGF, Gorham-Stout disease

## Abstract

Heterotopic ossification (HO) is a debilitating condition that commonly occurs after musculoskeletal injury and is characterized by the formation of bone in soft tissues. Despite advances in understanding its pathogenesis, effective therapies to reverse established heterotopic bone remain lacking. While lymphatic vessels are associated with the destruction of bone in rare diseases such as Gorham-Stout disease, their effect on HO has not been widely explored. Here, we use transgenic mice to determine whether targeted expression of the lymphatic growth factor VEGF-D can promote the therapeutic resorption of bone in a mouse model of HO. We show that control mice lack lymphatic vessels in heterotopic bone. In contrast, *Vegfd*-overexpressing (*Vegfd-OE*) mice develop lymphatic vessels in heterotopic bone and form significantly less heterotopic bone than control mice. Additionally, we demonstrate that VEGF-D overexpression or local delivery promotes the therapeutic resorption of established heterotopic bone. Mechanistically, we show that the transition of myeloid cells to osteoclasts and osteoclast-mediated bone resorption are enhanced in *Vegfd-OE* mice. These findings reveal a previously unrecognized role for lymphatic vessels in regulating heterotopic bone resorption and identify VEGF-D-mediated lymphangiogenesis as a promising therapeutic strategy for HO.

## Significance

Extremity musculoskeletal trauma is the most common injury pattern seen in humans. While in some instances, these musculoskeletal tissues heal, oftentimes tissue repair fails due to an aberrant cell fate program of the cells at the injury site. Heterotopic ossification (HO) is an example of this aberrant cell fate characterized by the abnormal differentiation of mesenchymal progenitor cells into chondrocytes and osteoblasts. Despite advances in understanding the pathogenesis of HO, effective therapies to reverse established heterotopic bone remain lacking. Here, we show that lymphangiogenesis (the growth of lymphatic vessels), which promotes the destruction of native bone in Gorham-Stout disease, can be harnessed to drive the therapeutic resorption of heterotopic bone in an animal model of HO.

Wound healing is a complex and essential biological process that restores tissue integrity after injury. Aberrant wound healing can result in severe complications such as heterotopic ossification (HO), which is characterized by the abnormal growth of bone in nonskeletal tissues (1). HO is a common complication following burns, extremity trauma and surgery, as well as spinal cord and traumatic brain injuries (1). Extremity trauma with bone fracture is a common form of injury and confers an increased risk of HO (2). The prevalence of HO is further increased in patients with a combination of musculoskeletal trauma and large surface-area burns, which is commonly seen in the setting of blast injuries (2). Importantly, patients with HO often have difficulty performing normal daily activities because they suffer from a decreased range of motion of involved joints and severe, chronic, debilitating pain (2). Although substantial progress has been made in understanding the underlying mechanisms of HO, it remains a challenging condition to treat. Current prophylactic approaches for traumatic HO (bisphosphonates, glucocorticoids, nonsteroidal anti-inflammatory drugs, and radiation therapy) have been met with limited success (3). Once HO develops, the standard of care is surgical resection. However, surgery rarely restores joint motion, and HO can recur following resection, posing a significant therapeutic challenge for patients (4). Therefore, there is an urgent need for new treatment strategies to prevent HO and resorb existing heterotopic bone lesions.

Lymphatic vessels play a crucial role in maintaining tissue fluid homeostasis, trafficking immune cells, and resolving inflammation after injury (5). Although lymphatic vessels are found in most regions of the body, bones have traditionally been shown to lack lymphatic vessels. Immunohistochemical staining of human and mouse bones revealed an absence of lymphatic vessels in bone (6–10). Unlike conventional histology, new tissue-clearing techniques render samples transparent, allowing an unprecedented three-dimensional (3D) view of structures. These new techniques have recently been used to characterize the 3D anatomy of the lymphatic vasculature surrounding bones. Surprisingly, authors of a recent tissue-clearing and 3D imaging study suggest that lymphatic vessels are present in healthy bone (11). However, two prior tissue-clearing and 3D imaging studies reported that lymphatic vessels do not reside in normal bone (12, 13). More recent scRNA-Seq and immunofluorescence experiments have also noted an absence of lymphatic vessels in bone (14–17). Thus, most experiments have concluded that regular bones do not contain lymphatic vessels. Although lymphatic vessels are not typically observed in healthy bones, they are routinely observed in the bones of patients with Gorham-Stout disease (18). Gorham-Stout disease is a lymphatic disease characterized by the growth of lymphatic vessels in bone and massive bone loss (19). In severe cases of the disease, it progresses until entire bones disappear (19). These findings establish that the growth of lymphatic vessels in bone is associated with bone resorption. However, whether lymphatic vessels promote the resorption of HO remains unclear.

In the present study, we use transgenic mice to determine whether the expression of vascular endothelial growth factor-D (VEGF-D) by *Hoxa11*-expressing cells promotes the therapeutic resorption of bone in trauma-induced HO. VEGF-D is a lymphatic growth factor that stimulates lymphangiogenesis by activating VEGFR3 on lymphatic endothelial cells (20), and *Hoxa11* is a transcription factor expressed by mesenchymal progenitor cells (MPCs) in the periosteum, endosteum, and tendon that form normal bone during development and heterotopic bone after injury (21, 22). We chose to express VEGF-D in the *Hoxa11* lineage because *Hoxa11* expression is spatially restricted to the zeugopod (radius/ulna and tibia/fibula) (21, 22). This anatomical specificity allows us to target VEGF-D overexpression to the precise region where HO develops in our injury model, thereby avoiding widespread VEGF-D expression throughout the body. We show that *Vegfd*-overexpressing (*Vegfd-OE*) mice develop heterotopic bone filled with lymphatic vessels. We also demonstrate that HO is significantly attenuated in *Vegfd*-*OE* mice. Moreover, we report that VEGF-D overexpression or local delivery triggers the resorption of established heterotopic bone. We also show that osteoclast-mediated bone resorption is increased in *Vegfd*-*OE* mice. Together, our findings suggest that site-directed lymphangiogenesis may serve as a preventive strategy for HO and facilitate the resorption of established heterotopic bone.

## Results

### Vegfd Induces the Resorption of Native Bone.

**Fig. 1.**
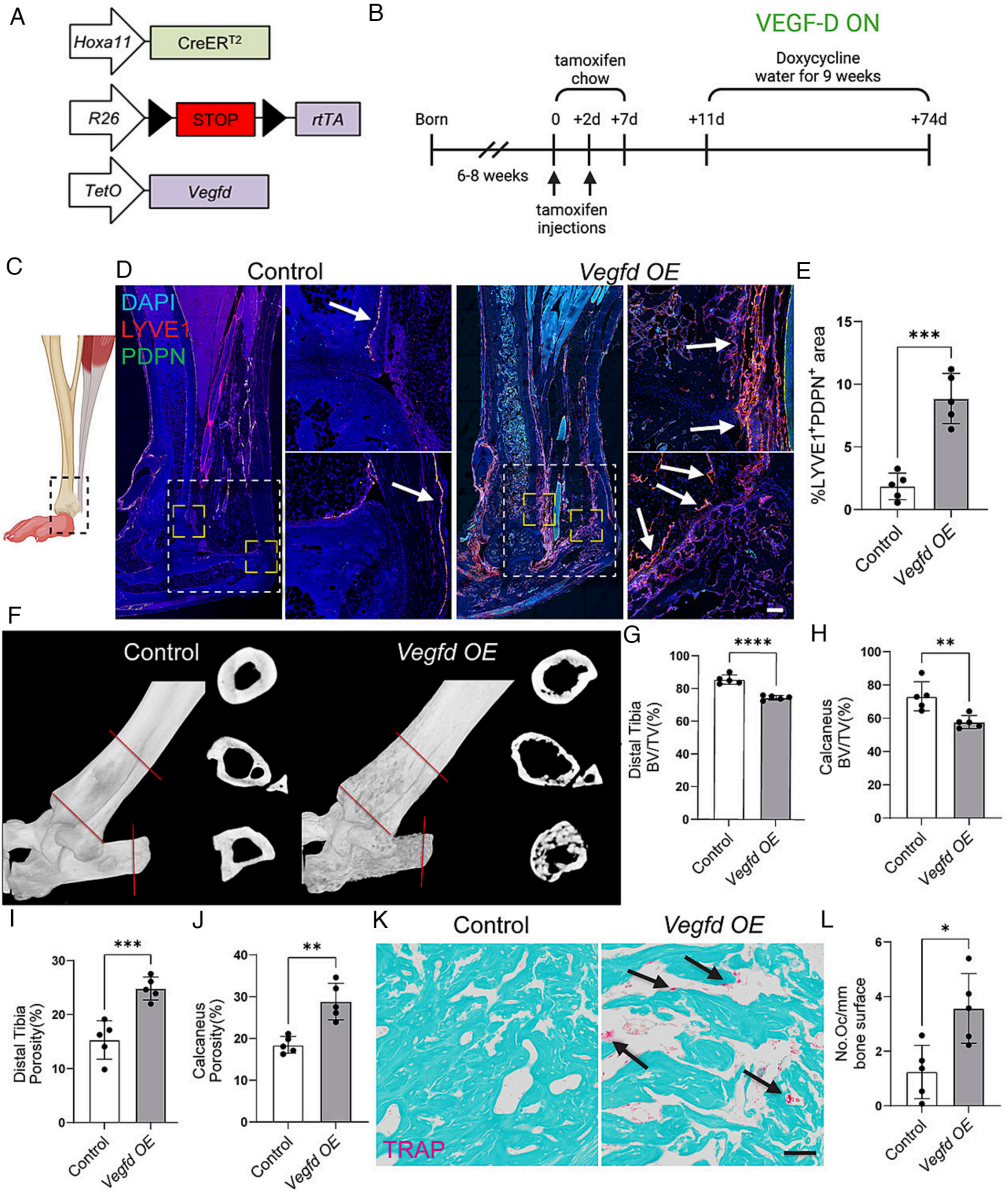
VEGF-D overexpression in uninjured limbs induces lymphangiogenesis and bone resorption. (*A*) Schematic of the inducible transgenic system. Following exposure of mice to tamoxifen, doxycycline drives the expression of VEGF-D by *Hoxa11*-lineage cells. (*B*) Schematic of the experimental timeline. Tamoxifen was administered to mice at 6 to 8 wk of age, followed by 9 wk of doxycycline water. (*C*) Schematic of the hindlimb showing the region of analysis. (*D*) Immunofluorescence staining of uninjured hindlimb tissue from control and *Vegfd*-overexpressing (OE) mice shows increased LYVE1^+^PDPN^+^ lymphatic vessels (red) in *Vegfd*-*OE* mice. DAPI (blue) stains the nuclei. White arrows point to lymphatic vessels. The dashed box marks the region of analysis. (*E*) Quantification of the LYVE1^+^PDPN^+^ area as a percentage of the total tissue area. (*F*) Representative µCT reconstructions of distal tibia and calcaneus from control and *Vegfd*-*OE* mice. (*G*–*H*) Bone volume to total volume (BV/TV) analysis shows decreased bone mass in the distal tibia (*G*) and calcaneus (*H*) of *Vegfd-OE* mice. (*I* and *J*) Quantification of cortical bone porosity in distal tibia (*I*) and calcaneus (*J*) shows increased porosity in *Vegfd-OE* mice. (*K*) TRAP-stained sections of the distal tibia show increased osteoclast numbers (arrows) in *Vegfd OE* mice. (*L*) Quantification of TRAP^+^ osteoclasts per bone surface area. Data are shown as mean ± SD. Unpaired Student’s *t* test. *P < 0.05, **P < 0.01, ***P < 0.001, ****P < 0.0001. Each point on the graph corresponds a data point collected from a single mouse (n = 5 mice/group). [Scale bars, 50 µm (panel *C*), 100 µm (panel *K*).]

Before investigating the effect of VEGF-D on HO, we first examined its effect on the maintenance of native bone. To this end, we generated mice that overexpress VEGF-D in *Hoxa11*-expressing cells (*Hoxa11^wt/CreERT2^;R26^wt/LSL-rtTA^;TetO-Vegfd* mice; referred to as *Vegfd*-*OE* mice for short). In our mouse model, *Hoxa11*-lineage cells express VEGF-D following exposure to tamoxifen and doxycycline (Fig. 1*A*). When *Vegfd*-*OE* mice and control littermates were 6 to 8 wk old, we administered tamoxifen to induce CreER^T2^-mediated recombination and expression of a reverse tetracycline transactivator (rtTA) in *Hoxa11*-expressing cells. After maintaining the mice on doxycycline for 9 wk, we collected their hindlimbs for analysis (Fig. 1 *B* and *C*). To assess the impact of VEGF-D expression on lymphatic vessels, we immunostained longitudinal sections of hindlimbs from control and *Vegfd-OE* mic. In control mice, lymphatic vessels were restricted to regions outside the bone and tendon (Fig. 1*D*). In contrast, there was a marked expansion of the lymphatic network in *Vegfd*-*OE* mice, with vessels present in the calcaneus (heel bone), distal tibia (the region near the ankle), and tendon (Fig. 1 *D* and *E*). Next, we examined the integrity of the distal tibia and calcaneus in control and *Vegfd-OE* mice by μCT imaging (Fig. 1*F*). We found that the Bone Volume/Tissue Volume (BV/TV) values for *Vegfd*-*OE* mice were significantly lower than those for control mice (Fig. 1 *G* and *H*). Furthermore, the porosity of bones was significantly greater in *Vegfd*-*OE* mice than in control mice (Fig. 1 *I* and *J*). Given these changes, we stained samples for tartrate-resistant alkaline phosphatase (TRAP) activity to identify osteoclasts (Fig. 1*K*). Osteoclasts are large multinucleated cells that resorb bone. We found that *Vegfd*-*OE* mice had significantly more osteoclasts than control mice (Fig. 1*L*). Together, these data suggest that overexpression of *Vegfd* in *Hoxa11*-lineage cells stimulates the formation of lymphatic vessels in native bone and osteoclast-mediated bone resorption.

### Vegfd Suppresses the Formation of Heterotopic Bone.

**Fig. 2.**
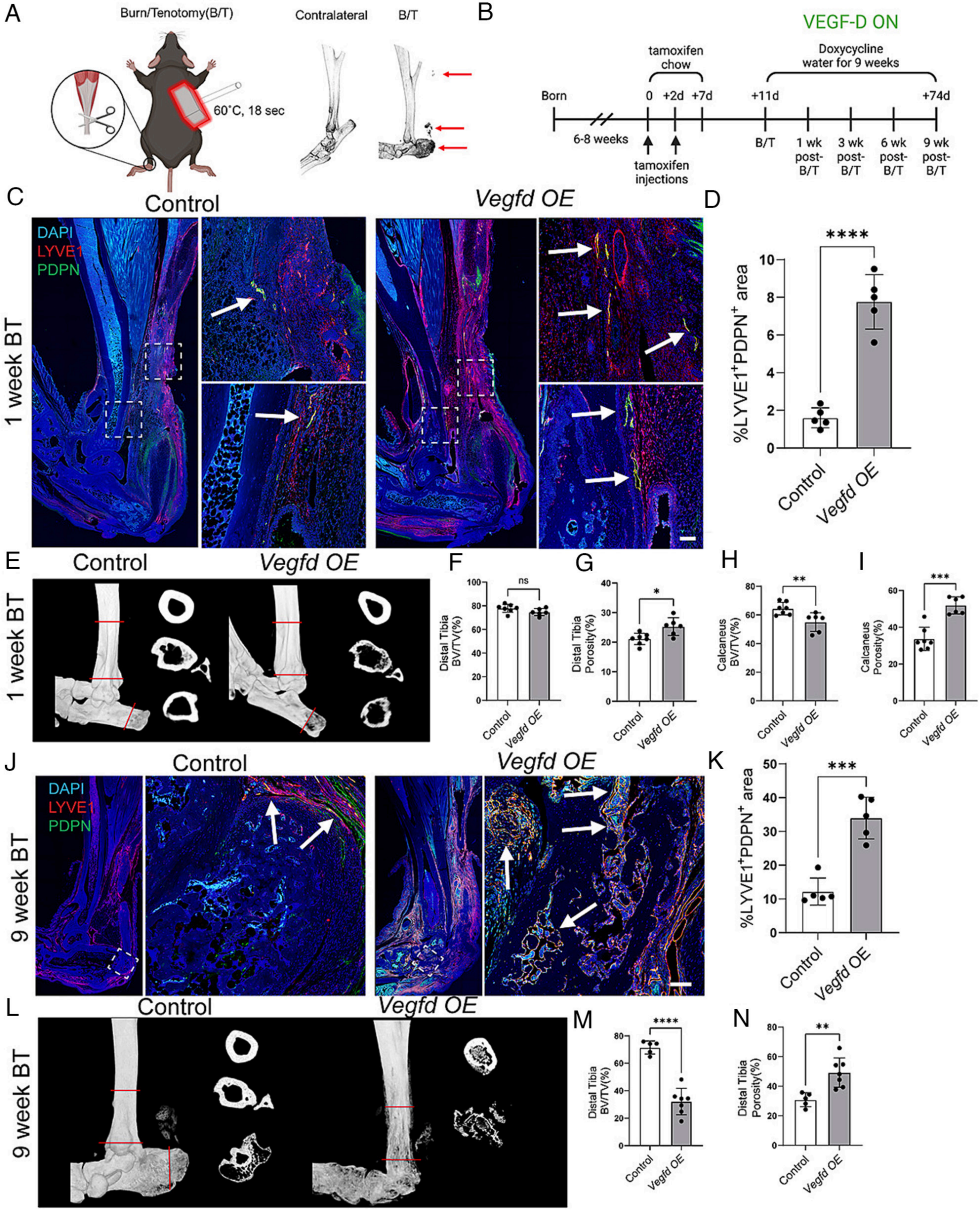
VEGF-D overexpression stimulates lymphangiogenesis and bone resorption following trauma-induced HO. (*A*) Schematic of the Burn/Tenotomy (B/T) model of HO. µCT images are included of injured (B/T) and uninjured (contralateral) limbs. The arrows point to heterotopic bone. (*B*) Schematic of the experimental timeline. Tamoxifen was given before the B/T injury, followed by a 9-wk course of doxycycline to promote VEGF-D expression. Tissue samples were collected 1, 3, 6, and 9 wk after the B/T injury. (*C*) Representative images showing LYVE1 (red), PDPN (green), and DAPI (blue) staining in injured limbs 1 wk post-B/T. (*D*) Quantification of the LYVE1^+^ PDPN^+^ area as a percentage of total tissue area 1 wk post-B/T. (*E*) Representative µCT images of calcaneus and tibia 1 wk post-B/T. (*F* and *G*) Quantification of bone volume to total volume (BV/TV) in the distal tibia (*F*) and calcaneus (*G*) reveals mild bone loss in *Vegfd-OE* mice. (*H* and *I*) Cortical porosity is significantly increased in the distal tibia (*H*) and calcaneus (*I*) of *Vegfd-OE* mice at 1-wk post-B/T. (*J*) Representative images showing LYVE1 (red), PDPN (green), and DAPI (blue) staining 9 wk post-B/T in control and *Vegfd-OE* mice. (*K*) Quantification of the LYVE1^+^ PDPN^+^ area as a percentage of the total tissue area 9 wk post-B/T. (*L*) Representative µCT images of the tibia 9 wk post-B/T in control and *Vegfd-OE* mice. (*M* and *N*) Significant reduction in BV/TV and increased cortical porosity the distal tibia of *Vegfd OE* mice indicates progressive bone resorption. Data are shown as mean ± SD. Unpaired Student’s *t* test. *P < 0.05, **P < 0.01, ***P < 0.001, ****P < 0.0001. Each point on the graph corresponds a data point collected from a single mouse (n = 5 to 7 mice/group). [Scale bar, 50 µm (panels *C* and *J*).]

To determine whether lymphatic vessels influence heterotopic bone, we utilized our previously developed mouse model of trauma-induced HO (23). In this model, mice gradually develop heterotopic bone in their Achilles tendon (floating HO) and around their calcaneus (bone associated HO) after receiving an Achilles tendon transection and a 30% total body surface area burn (Burn/tenotomy (B/T) model) (Fig. 2*A*). When *Vegfd-OE* mice and control littermates were 6 to 8 wk old, we injected them with tamoxifen to induce CreER^T2^-mediated recombination in *Hoxa11-*positive cells. After a 4-d washout period, we performed the B/T procedure on the mice and then gave them doxycycline drinking water. After maintaining the mice on doxycycline drinking water for 1, 3, 6, or 9 wk, we collected the injured hindlimbs for analysis (Fig. 2*B*). As before, we characterized changes to lymphatic vessels by immunostaining longitudinal hindlimb sections and changes to bone by μCT.

one-weeks postinjury, lymphatic vessels in control mice were restricted to regions outside the bone and tendon (Fig. 2*C*). However, *Vegfd-OE* mice exhibited a significant increase in the area of lymphatic vessels at the injury site, with lymphatic vessels beginning to invade the tendon and growing within the injured site (Fig. 2*D*). We then characterized bone structure in control and *Vegfd-OE* mice and found that the distal tibia BV/TV appeared similar between the two groups (Fig. 2 *E* and *F*). However, the porosity was increased in distal tibia of *Vegfd-OE* mice (Fig. 2*G*). Notably, the calcaneus began to resorb in *Vegfd-OE* mice, with *Vegfd-OE* mice calcaneal bone showing lower BV/TV and greater porosity compared to control (Fig. 2 *H* and *I*). Consistent with previous B/T experiments (14), there were no signs of HO in control or *Vegfd*-*OE* mice 1-wk postinjury.

Three weeks postinjury, and higher porosity values (*SI Appendix,* Fig. S1 *F* and *G*). lymphatic vessels had invaded the tendon in control mice (*SI Appendix,* Fig. S1*A*). However, they remained outside the calcaneus and distal tibia (*SI Appendix,* Fig. S1*A*). In contrast, the lymphatic network significantly expanded at the injury site in *Vegfd-OE* mice, with lymphatic vessels having invaded the tendon, calcaneus, and distal tibia (*SI Appendix,* Fig. S1*B*). The bone structure of the distal tibia also started to appear different between control and *Vegfd*-*OE* mice (*SI Appendix,* Fig. S1*C*). The BV/TV values for the distal tibia were significantly lower in *Vegfd-OE* mice compared to control mice (*SI Appendix,* Fig. S1*D*). Moreover, the porosity in the distal tibia was markedly higher in *Vegfd*-*OE* mice than in control mice (*SI Appendix,* Fig. S1*E*). While control mice began to develop heterotopic bone around the calcaneus, *Vegfd-OE* displayed continued resorption of the calcaneus, resulting in significantly lower calcaneal BV/TV values

Six weeks postinjury, lymphatic vessels were observed in the tendons of control mice but remained outside the native and heterotopic bone (*SI Appendix,* Fig. S1*H*). In *Vegfd-OE* mice, lymphatic vessels continued to grow in the tendon, calcaneus, and distal tibia (*SI Appendix,* Fig. S1*H*). Floating heterotopic bone in the tendon of *Vegfd-OE* mice also contained lymphatic vessels (*SI Appendix,* Fig. S1*I*). The distal tibia in *Vegfd*-*OE* mice continued to resorb, resulting in significantly lower BV/TV values and higher porosity values in *Vegfd*-*OE* mice than in control mice (*SI Appendix,* Fig. S1 *J*–*L*). While heterotopic bone continued to form around the calcaneus in control mice, the extensive calcaneal resorption in *Vegfd*-*OE* mice limited our ability to make μCT measurements (*SI Appendix,* Fig. S1*J*).

**Fig. 3.**
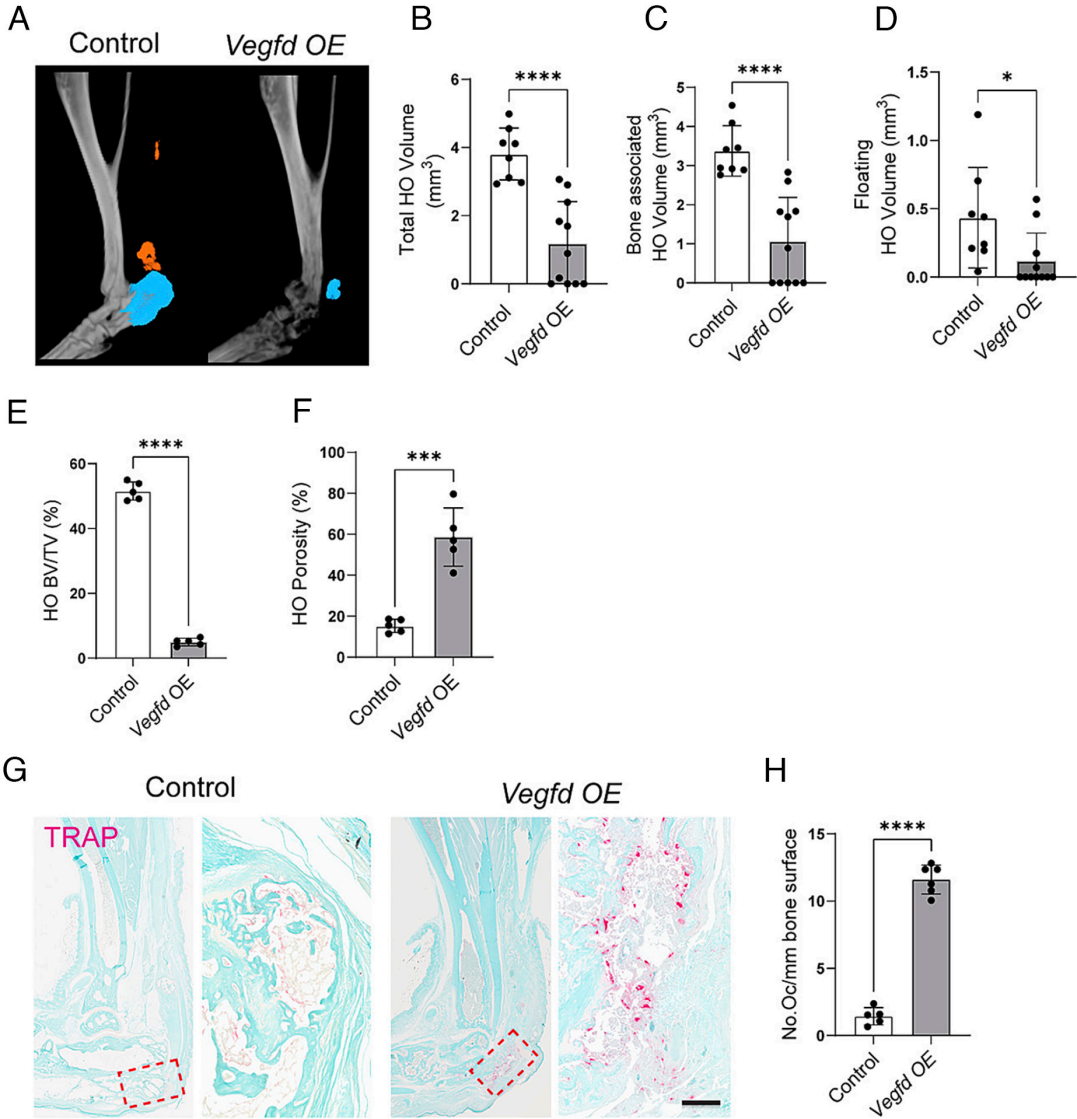
VEGF-D overexpression enhances osteoclast activity and reduces the volume of HO. (*A*) Representative 3D μCT reconstructions showing segmented HO volume (colored) in the hindlimbs of control and *Vegfd-OE* mice 9 wk post-B/T. (*B*–*D*) Quantification of (*B*) total HO volume, (*C*) bone-associated HO volume, and (*D*) floating HO volume. All measures are significantly reduced in *Vegfd-OE* mice. (*E* and *F*) Quantification of HO (BV/TV) (*E*) and HO porosity (%) (*F*) further confirms reduced HO and increased bone resorption in the *Vegfd OE* group. (*G*) Representative images of TRAP-stained histological sections from control and *Vegfd-OE* mice 9 wk post-B/T. Arrows highlight TRAP^+^ osteoclasts in red, indicating increased osteoclast activity in *Vegfd-OE* samples. (*H*) Quantification of TRAP^+^ osteoclasts per millimeter of bone surface reveals a significant increase in osteoclasts in *Vegfd-OE* mice. Data are shown as mean ± SD. Unpaired Student’s *t* test. *P < 0.05, ***P < 0.001, ****P < 0.0001. Each point on the graph corresponds a data point collected from a single mouse (n = 5 to 11 mice/group). [Scale bar, 100 µm (panel *G*).]

Nine weeks postinjury, lymphatic vessels persisted in the tendons of control mice (Fig. 2*J*). However, no lymphatic vessels were in the native or heterotopic bone of control mice (Fig. 2*J*). In contrast, the lymphatic network grew in *Vegfd-OE* mice, and they exhibited lymphatic vessels within their native bone, the injured tendon, and heterotopic bone (Fig. 2 *J* and *K*). Although we observed a strong lymphangiogenic response in *Vegfd-OE* mice, we did not observe a statistically significant difference in blood vessel density at the injury site between control and *Vegfd-OE* mice (*SI Appendix,* Fig. S2 *H* and *I*). *Vegfd-OE* mice exhibited lower BV/TV values and higher porosity values in the distal tibia compared to control mice (Fig. 2 *L*–*N*). Moreover, *Vegfd*-*OE* mice had significantly less heterotopic bone than control mice (Fig. 3*A*). The total HO volume, bone-associated HO volume, floating HO volume, and HO BV/TV values were all significantly lower in *Vegfd-OE* mice than in control mice (Fig. 3 *B*–*E*). Additionally, the porosity of the heterotopic bone was significantly greater in *Vegfd*-*OE* mice than in control mice (Fig. 3*F*). Consistent with *Vegfd-OE* mice exhibiting more bone resorption than control mice, we found that *Vegfd*-*OE* mice had significantly more osteoclasts in their heterotopic bone (Fig. 3 *G* and *H*). These data suggest that local VEGF-D overexpression induces the gradual invasion of native and heterotopic bone by lymphatic vessels and the resorption of regional native and heterotopic bone.

### Injury Enhances the Phenotype of Vegfd-OE Mice.

During our studies, we noticed that injured *Vegfd*-*OE* limbs (e.g., Fig. 2*L*) displayed a significantly more severe phenotype than uninjured *Vegfd-OE* limbs (e.g., Fig. 1*F*). This observation prompted us to closely compare injured *Vegfd-OE* limbs to contralateral uninjured *Vegfd-OE* limbs 9 wk postinjury. We found that the lymphatic vessel area was significantly greater in the injured than in the uninjured limbs of *Vegfd*-*OE* mice (*SI Appendix,* Fig. S2 *A* and *B*). Additionally, the injured limbs of *Vegfd*-*OE* mice exhibited significantly more resorption of the distal tibia than uninjured limbs (*SI Appendix,* Fig. S2 *C* and *D*). Consistent with injured limbs in *Vegfd-OE* mice exhibiting more bone resorption, there were significantly more osteoclasts in the injured than in the uninjured limbs of *Vegfd*-*OE* mice (*SI Appendix,* Fig. S2*F*). These results suggest that injury enhances the phenotype in our *Vegfd*-*OE* model.

### Hoxa11-Lineage Cells Expand After Injury and Do Not Adopt an LEC Fate.

Next, we set out to investigate why injured limbs exhibit a more severe phenotype than uninjured limbs in *Vegfd*-*OE* mice. Previous studies have shown that *Hoxa11^CreERT2^*-labeled cells expand after injury (21, 22). An increase in *Hoxa11^CreERT2^*-labeled cells in injured limbs would increase the number of VEGF-D-expressing cells in our mouse model. To determine whether the population of *Hoxa11^CreERT2^*-labeled cells expands following the B/T procedure, we used a *TetO-H2B::eGFP* reporter to lineage trace *Hoxa11*-expressing cells. *TetO-H2B::eGFP* mice express a green fluorescent protein (GFP) tagged version of histone H2B in a doxycycline-regulatable manner (*SI Appendix,* Fig. S3*A*). After several rounds of breeding, we generated *Hoxa11^wt/CreERT2^;R26^wt/LSL-rtTA^;TetO-H2B::eGFP* mice (referred to as *eGFP*-*OE* mice for short) and control littermates. When mice were 6 to 8 wk old, we injected them with tamoxifen to induce CreER^T2^-mediated expression of rtTA in *Hoxa11*-expressing cells. After a washout period of 4 d, we performed the B/T procedure on the mice and then gave them doxycycline drinking water for 9 wk (*SI Appendix,* Fig. S3*B*). We then analyzed GFP expression in longitudinal sections of injured and uninjured limbs from *eGFP-OE* mice. We found that the injured limbs of *eGFP-OE* mice had significantly more GFP-expressing cells than uninjured limbs (*SI Appendix,* Fig. S3 *C* and *D*). These results suggest that the *Hoxa11*-labeled cell population expands after injury.

Collagen 2 (Col2) is highly expressed by chondrocytes and is important for endochondral ossification (24). A recent lineage-tracing study found that Col2-positive cells differentiate into LECs instead of chondrocytes or osteoblasts in a mouse model of HO (24). Because *Hoxa11* is expressed by MPCs that give rise to chondrocytes and osteoblasts (21, 22), we examined limbs from control and *eGFP-OE* mice to determine whether *Hoxa11*-expressing MPCs differentiate into LECs instead of chondrocytes and osteoblasts. We focused our analysis on samples collected 9 wk post-B/T. Notably, we did not observe colocalization between GFP and LYVE1 in the injury site or around heterotopic bone in *eGFP-OE* mice (*SI Appendix,* Fig. S3 *C*, *Inset*). These findings suggest that *Hoxa11*-expressing MPCs do not adopt an LEC fate in our model of trauma-induced HO. We also analyzed heterotopic bone in control and *eGFP-OE* mice by μCT. We found that the amount of heterotopic bone was not significantly different between the two groups of mice (*SI Appendix,* Fig. S3 *E*–*J*). These results suggest that simply overexpressing a transgene in the *Hoxa11* lineage does not impair HO.

### Vegfd Overexpression Indirectly Induces Osteoclastogenesis.

**Fig. 4.**
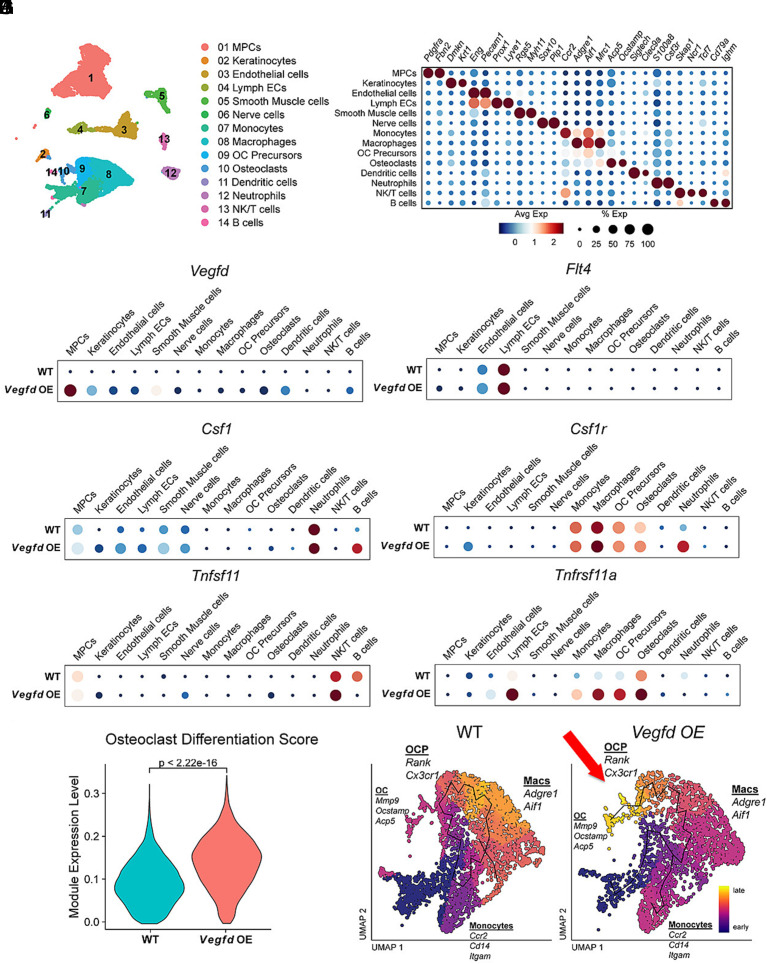
Single-cell RNA-seq reveals transcriptional changes in osteoclast precursors and differentiation pathways in *Vegfd OE* mice. (*A*) UMAP clustering of pooled single-cell RNA-seq data from wild-type (WT) and *Vegfd-OE* hindlimb tissues identifies 14 distinct cell populations, including mesenchymal progenitor cells (MPCs), keratinocytes, endothelial cells, lymphatic endothelial cells (LECs), immune cell subsets, and osteoclast precursors (OC precursors). (*B*) Dot plot showing average expression (color intensity) and percentage of cells expressing key cluster-defining genes (dot size) across identified cell types. (*C*–*H*) Dot plots of selected genes involved in lymphangiogenesis and osteoclast biology across cell types and genotypes (WT vs *Vegfd-OE*). (*I*) Violin plot showing a significant increase in osteoclast differentiation module scores in *Vegfd-OE* mice (*P* < 2.2e–16, Wilcoxon rank-sum test). (*J*) UMAP plots highlighting the osteoclast lineage trajectory. OC precursors and mature osteoclasts (marked by *Cx3cr1*, *Rank*, *Acp5*, *Atp6v0d2*) are shown across the UMAP. Pseudotime trajectory analysis reveals increased representation of late-stage osteoclasts in *Vegfd-OE* mice (red arrow).

We next performed single-cell RNA sequencing (scRNA-seq) to investigate the molecular mechanisms by which VEGF-D overexpression affects HO. We collected tissue from the injury site 1 wk after performing the B/T procedure on *Vegfd-OE* mice and submitted these samples for scRNA-seq. We compared these data to a scRNA-Seq dataset for wild-type mice 7 d post-B/T (gene expression omnibus; GSE126060). Unsupervised clustering revealed 14 distinct cell populations within the injured tissue (Fig. 4*A*). These populations include: MPCs (*Pdgfra^+^*, *Fbn2^+^*), keratinocytes (*Dmkn^+^, Krt1^+^*), endothelial cells (*Eng^+^, Pecam1^+^*), lymphatic endothelial cells (*Prox1^+^, Lyve1^+^*), smooth muscle cells (*Rgs5^+^, Myh11^+^*), nerve cells (*Sox10^+^, Plp^+^*), monocytes (*Ccr2^+^, Aif^+^*), macrophages (*Adgre1^+^, Aif1^+^, Mrc1^+^*), osteoclast precursors (*Aif1^+^, Acp5^+^*), osteoclasts (*Acp5^+^, Ocstamp^+^*), dendritic cells (*Siglech^+^, Clec9a^+^*), neutrophils (*S100a8^+^, Csf3r^+^*), NK/T cells (*Skap1^+^, Ncr1^+^, Tcf7^+^*), and B cells (*Cd79a^+^, Ighm^+^*) (Fig. 4*B*).

We first assessed the expression pattern of *Vegfd* in our dataset. We found that *Vegfd* was dramatically higher in *Vegfd-OE* mice than in control mice, with MPCs exhibiting the greatest expression (Fig. 4*C*). This result is consistent with our *Vegfd-OE* model, where *Hoxa11*-expressing MPCs drive the expression of *Vegfd*. We also compared our scRNA-Seq data for injured limbs to that of uninjured limbs from *Vegfd-OE* mice. This analysis revealed that *Vegfd* was significantly higher in injured than in uninjured limbs of *Vegfd-OE* mice (*SI Appendix,* Fig. S2*E*). This finding aligns with our finding that the *Hoxa11*-lineage expands in mice after injury (*SI Appendix,* Fig. S3 *C* and *D*). We next evaluated the expression of *Flt4*, which encodes for the VEGF-D receptor VEGFR3. *Flt4* was most abundant in lymphatic endothelial cells and moderately expressed by blood endothelial cells in control and *Vegfd-OE* mice (Fig. 4*D*). *Flt4* was not expressed by myeloid cells, osteoclast precursors, or osteoclasts (Fig. 4*D*).

Next, we examined the expression of genes that regulate osteoclast development. Osteoclasts differentiate from myeloid progenitor cells through a series of steps driven by macrophage colony-stimulating factor (M-CSF/*Csf1*) and receptor activator of nuclear factor-κB ligand (RANKL/*Tnfsf11*), which bind to the receptors CSF1R (*Csf1r*) and RANK (*Tnfrsf11a*), respectively (25). We found that neutrophils and B cells highly expressed *Csf1*, whereas *Tnfsf11* was primarily expressed by NK/T cells in *Vegfd-OE* mice (Fig. 4 *G* and *E*). Lymphatic endothelial cells in *Vegfd-OE* mice did not express appreciable levels of either factor (Fig. 4 *G* and *E*). *Csfr1* was expressed by monocytes, macrophages, neutrophils, OC precursors, and osteoclasts in control and *Vegfd-OE* mice (Fig. 4*F*). Interestingly, *Tnfrsf11a* (RANK) was higher in lymphatic endothelial cells, monocytes, macrophages, OC precursors, and osteoclasts in *Vegfd-OE* mice than in control mice (Fig. 4*H*). To further investigate the impact of VEGF-D on osteoclastogenesis, we analyzed the expression of osteoclast differentiation genes by monocytes, macrophages, OC precursors, and osteoclasts in control and *Vegfd-OE* mice to generate osteoclast differentiation scores. We found that osteoclast differentiation scores were significantly increased in *Vegfd-OE* mice compared with control mice (Fig. 4*I*), indicating enhanced myeloid fate commitment toward the osteoclast lineage. Consistent with this finding, pseudotime trajectory analysis revealed a distinct branching trajectory toward terminally differentiated osteoclasts in *Vegfd-OE* mice that was absent in control mice (Fig. 4*J*). Although trajectory analysis revealed differences in osteoclast differentiation between control and *Vegfd-OE* mice, it did not reveal any significant differences in the differentiation of MPCs toward osteoblast or chondrocyte lineages between control and *Vegfd-OE* mice (*SI Appendix,* Fig. S4 *C*–*F*).

To determine whether VEGF-D directly regulates osteoclast differentiation, bone marrow–derived monocytes/macrophages were treated with VEGF-D during an in vitro osteoclast fusion assay; however, no significant differences were observed in the number of TRAP^+^ multinucleated osteoclasts compared with control conditions (*SI Appendix,* Fig. S4 *A* and *B*). In addition, to assess whether VEGF-D directly influences osteogenic differentiation, osteoprogenitor cells were cultured in osteogenic differentiation medium (ODM) supplemented with increasing concentrations of VEGF-D (1, 10, or 100 ng/ml). Neither alkaline phosphatase staining nor ALP activity differed significantly between VEGF-D-treated groups and ODM controls, indicating no effect on early osteogenic differentiation. Similarly, Alizarin Red S staining and quantification demonstrated comparable mineralization across all conditions, suggesting that VEGF-D does not directly affect late-stage osteogenic differentiation in vitro (*SI Appendix,* Fig. S4 *G*–*I*).

### Vegfd Induces the Resorption of Existing HO.

**Fig. 5.**
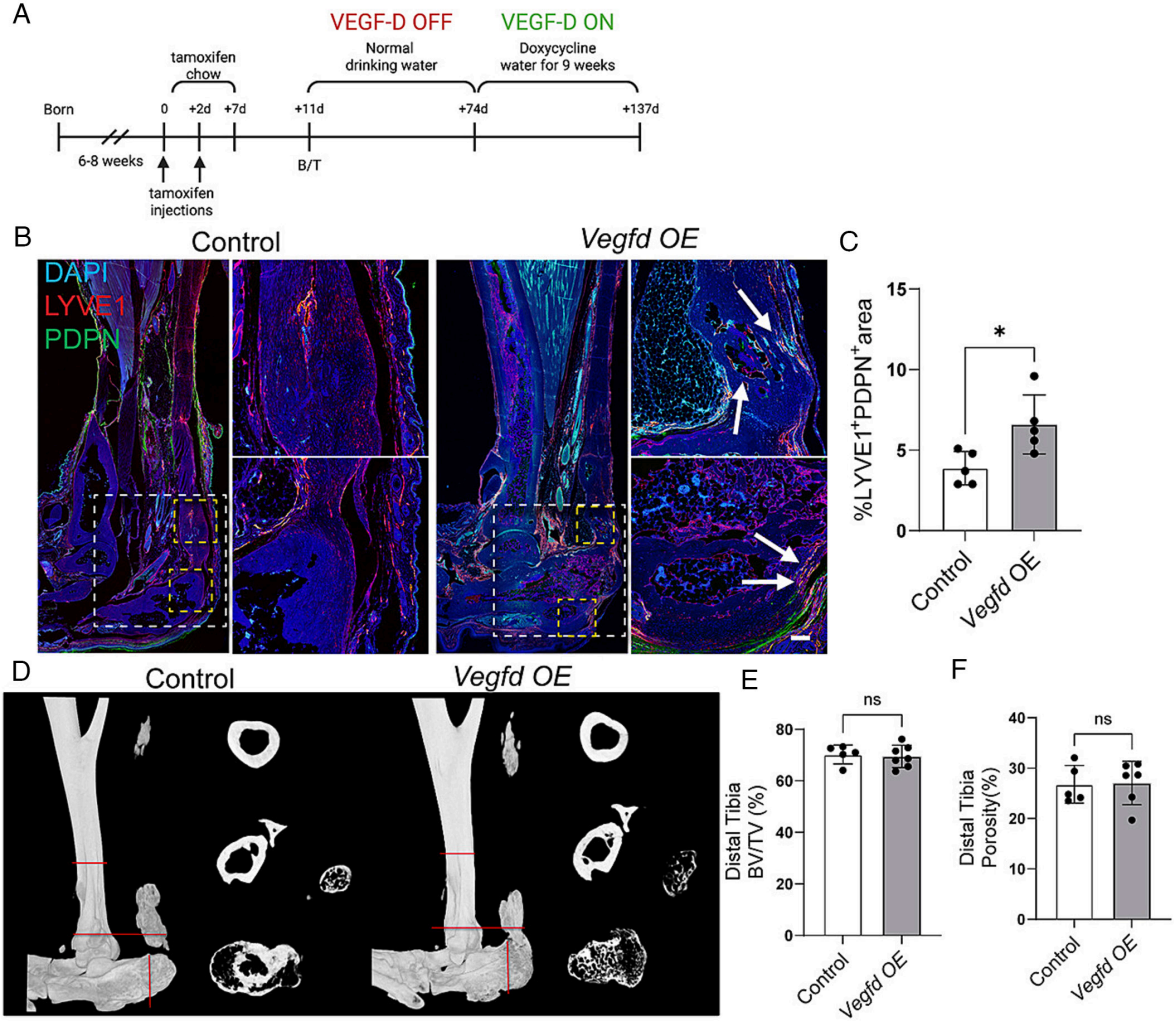
Late VEGF-D overexpression increases lymphatic vessel formation and promotes HO resorption without affecting native bone. (*A*) Schematic of the experimental timeline. Mice were administered tamoxifen to induce recombination, followed by B/T injury. VEGF-D expression was induced with doxycycline 74 d post-B/T. Mice were maintained on doxycycline drinking water for 9 wk. (*B*) Representative images of longitudinal hindlimb sections from control and *Vegfd OE* mice showing LYVE1^+^ (red) PDPN^+^ (green) and DAPI (blue) staining. Insets show magnified views of boxed regions. (*C*) Quantification of LYVE1^+^ PDPN^+^ area as a percentage of total tissue area (boxed regions) reveals a significant increase in lymphatic vessels in *Vegfd-OE* mice within the injured tissue. (*D*) Representative μCT images of the distal tibia and adjacent HO tissue in control and *Vegfd-OE* mice. Red brackets indicate regions analyzed for bone morphometry. (*E* and *F*) Quantification of HO parameters shows significantly decreased (*E*) BV/TV and (*F*) porosity in *Vegfd-OE* mice compared to controls. Data are shown as mean ± SD. Unpaired Student’s *t* test. ns = not significant, **P < 0.01. Each point on the graph corresponds a data point collected from a single mouse (n = 5 to 7 mice/group). [Scale bar 50 µm (panel *B*).]

**Fig. 6.**
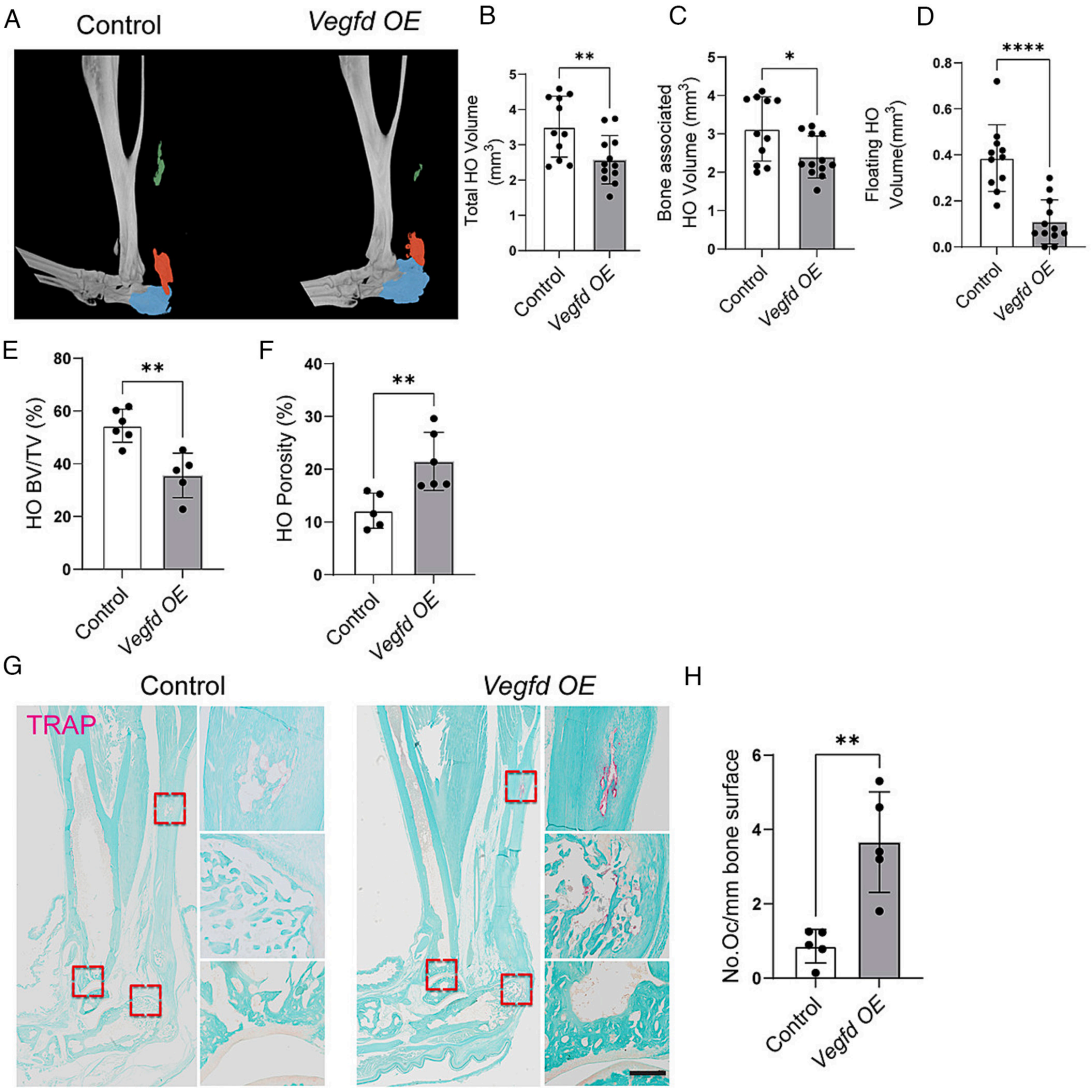
VEGF-D overexpression reduces existing HO and increases osteoclast numbers. (*A*) Representative 3D reconstructed μCT images of hindlimbs showing segmented HO in control and *Vegfd-OE* mice. HO regions are color-coded (blue = bone-associated HO; red and green = floating HO). (*B*–*D*) Quantification of HO volumes: (*B*) Total HO volume, (*C*) bone-associated HO volume, and (*D*) floating HO volume were all significantly reduced in *Vegfd-OE* mice compared to controls. (*E*) Bone volume/total volume (BV/TV) and (*F*) porosity values for control and *Vegfd*-*OE* mice. (*G*) Representative images of TRAP-stained hindlimb sections from control and *Vegfd-OE* mice. Red boxes highlight regions of interest shown at higher magnification. (*H*) Quantification of TRAP^+^ osteoclasts on HO bone surface (No. Oc/mm) shows significantly increased osteoclast numbers within HO in *Vegfd-OE* mice. Data are shown as mean ± SD. Unpaired Student’s *t* test. *P < 0.05, **P < 0.01, ****P < 0.0001. Each point on the graph corresponds a data point collected from a single mouse (n = 5 to 12 mice/group).

Next, we set out to determine whether lymphatic vessels could promote the resorption of established heterotopic bone. We administered tamoxifen to control and *Vegfd*-*OE* mice when they were 6 to 8 wk old and then performed the B/T procedure on them after a 4-d washout period. Mice were allowed to develop heterotopic bone for 9 wk, after which doxycycline was administered for an additional 9 wk (Fig. 5*A*). As before, control mice had lymphatic vessels in their tendon but lacked lymphatic vessels in their normal and heterotopic bone (Fig. 5*B*). In contrast, *Vegfd*-*OE* mice had lymphatic vessels in their tendon and floating heterotopic bone (Fig. 5 BLymphatic vessel area was significantly greater in *Vegfd-OE* mice compared to control mice (Fig. 5*C*). Although *Vegfd-OE* mice had lymphatics in their tendon and heterotopic bone, they lacked lymphatic vessels in adjacent normal bone (e.g., distal tibia) (*SI Appendix*, Fig. S5 *A* and *B*). We then assessed changes in bone structure by μCT (Fig. 5*D*). We found that the BV/TV and porosity values for the distal tibia were not significantly different between control and *Vegfd*-*OE* mice (Fig. 5 *E* and *F*). In contrast, *Vegfd-OE* mice exhibited significantly lower total HO volume, bone-associated HO volume, floating HO volume, and HO BV/TV values compared to control mice (Fig. 6 *A*–*E*). Additionally, heterotopic bone porosity was significantly greater in *Vegfd*-*OE* mice than in control mice (Fig. 6*F*). *Vegfd*-*OE* mice also had significantly more osteoclasts in their heterotopic bone than control mice (Fig. 6 *G* and *H*). These results suggest that late induction of VEGF-D spares native bone from damage and induces lymphatic invasion and osteoclast-mediated resorption of existing heterotopic bone.

### Exogenous VEGF-D Promotes Therapeutic HO Resorption.

**Fig. 7.**
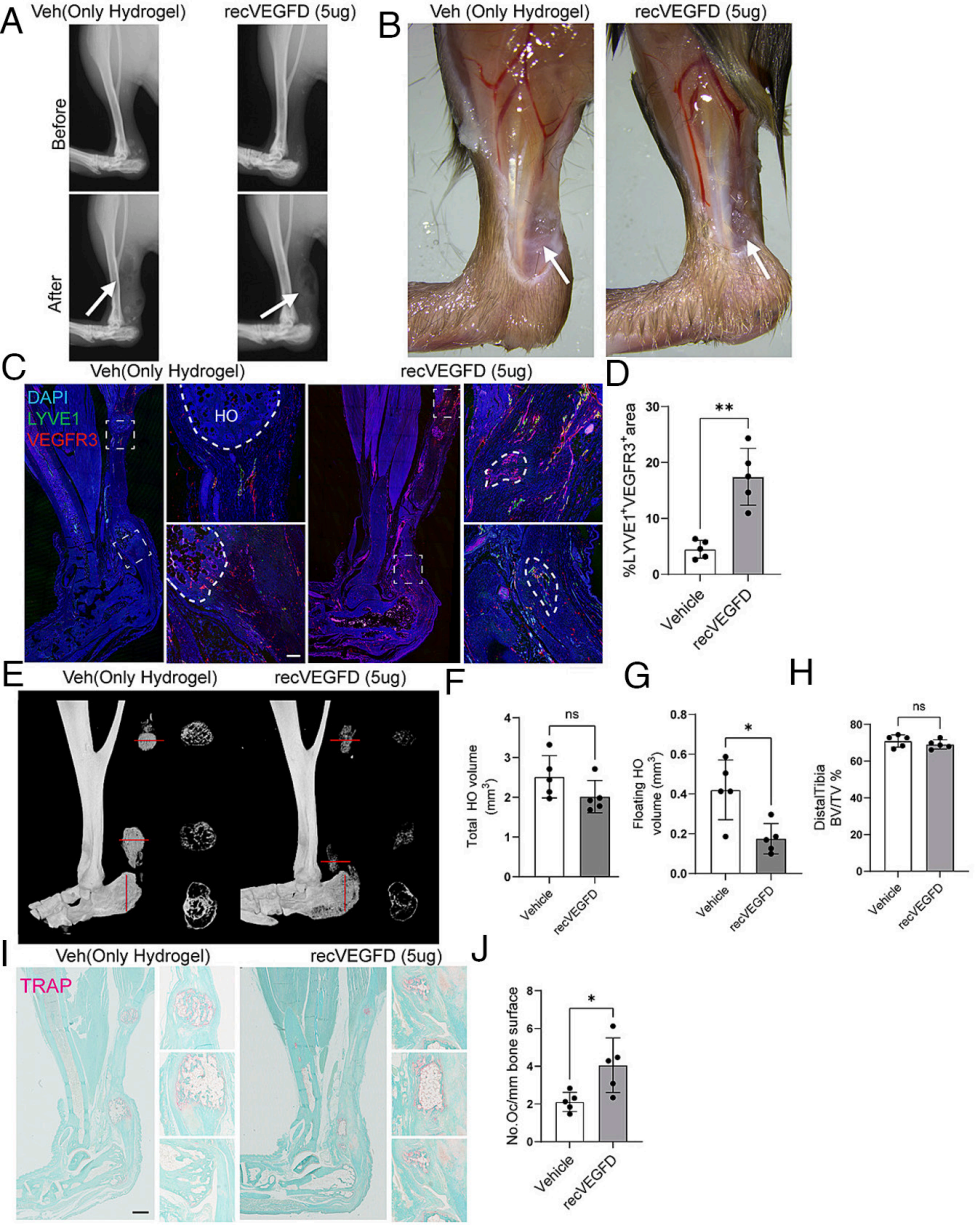
Local recombinant VEGF-D hydrogel delivery promotes lymphatic vessel invasion and resorption of mature heterotopic bone. (*A*) Representative radiographs of established HO lesions before and after implantation of vehicle (hydrogel alone) or recombinant VEGF-D-loaded hydrogel (5 µg of VEGF-D). Arrows indicate hydrogel implantation. (*B*) Representative gross images of hindlimbs following hydrogel implantation showing hydrogel implant at harvest day. (*C*) Representative immunofluorescence images of HO lesions stained for DAPI (blue), LYVE1 (green), and VEGFR3 (red) in vehicle and recombinant VEGF-D-treated limbs, demonstrating increased lymphatic vessel invasion following VEGF-D delivery. (*D*) Quantification of LYVE1^+^VEGFR3^+^ area within the HO region. (*E*) Representative µCT reconstructions of hindlimbs from vehicle- and recombinant VEGF-D-treated mice, with dotted area indicating HO lesions in magnified images. (*F*) Quantification of total HO volume, (*G*) floating HO volume, and (*H*) distal tibial bone volume fraction (BV/TV). (*I*) Representative TRAP-stained sections of HO lesions from vehicle and recombinant VEGF-D-treated mice. (*J*) Quantification of TRAP^+^ osteoclast number per HO region. Data are shown as mean ± SD. Unpaired Student’s t test. *P < 0.05, **P < 0.01. Each point on the graph corresponds a data point collected from a single mouse (n = 5 mice/group). [Scale bars, 50 µm (panel *C*) and 200 µm (panel *I*).]

To extend our overexpression studies, we set out to determine whether exogenous VEGF-D could promote the therapeutic resorption of established HO. We first performed the B/T procedure on wild-type C57BL/6 J mice and allowed them to form heterotopic bone for 7 wk. We then incorporated recombinant VEGF-D (5 µg) into an injectable slow-release hydrogel. The VEGF-D-containing or empty hydrogel (vehicle) was then implanted adjacent to the site of floating HO (Fig. 7 *A* and *B*). We found that VEGF-D-treated mice had significantly more lymphatics in the injury site than vehicle-treated mice (Fig. 7 *C* and *D*). Although total HO volume was reduced, it was not significantly different between VEGF-D- and vehicle-treated mice. However, VEGF-D-treated mice had significantly less floating HO than vehicle-treated mice (Fig. 7 *E*–*H*). Importantly, the VEGF-D-containing hydrogel was placed next to the floating HO, indicating it had a regional effect on HO resorption. VEGF-D-treated mice also had significantly more osteoclasts in their floating HO than vehicle-treated mice (Fig. 7 *I* and *J*). These experiments suggest that exogenous VEGF-D can induce lymphangiogenesis and promote the resorption of regional heterotopic bone.

## Discussion

**Fig. 8.**
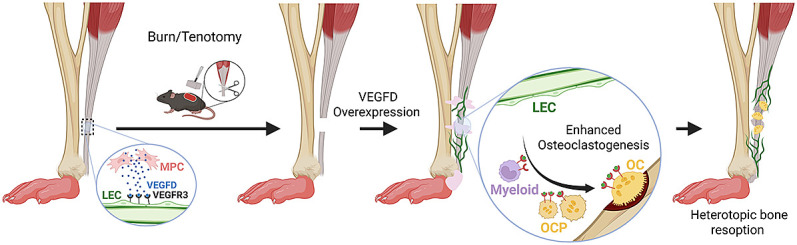
VEGF-D expression stimulates lymphatic vessel invasion of heterotopic bone, osteoclastogenesis, and heterotopic bone resorption.

HO remains a major clinical challenge following trauma, orthopedic surgery, or burns. Current therapies are limited, and once HO forms, reversal is exceedingly difficult. Lymphatic vessels have been associated with the destruction of native bone in diseases such as Gorham-Stout disease (19). However, the effect of lymphatic vessels on HO was previously unknown. In this study, we used *Vegfd*-*OE* mice to investigate the effect of site-directed lymphangiogenesis on trauma-induced HO. We found that VEGF-D expression stimulates lymphatic vessel invasion of heterotopic bone and osteoclast-mediated bone resorption (Fig. 8). Our findings suggest that site-directed lymphangiogenesis may serve as a treatment to prevent HO and promote the resorption of existing heterotopic bone. Furthermore, our study underscores how insights into the pathogenesis of rare diseases can lead to innovative treatments for more common conditions.

Recently, the anatomy of the lymphatic vasculature and bone has become controversial. One tissue-clearing and 3D imaging study suggested that lymphatic vessels are in healthy bone (11), whereas two others concluded that lymphatic vessels are absent from normal bone (12, 13). Immunohistochemical studies have also concluded that lymphatic vessels do not reside in bone (6, 7, 9, 10, 12, 14, 16, 17, 26). Those reports have found that lymphatic vessels are restricted to the connective tissue surrounding bones. We have found that native and heterotopic bone in wild-type mice lacks lymphatic vessels. Thus, our findings align with most reports on the anatomy of the lymphatic vasculature and bone (6, 7, 9, 10, 12–14, 26).

Notably, we found that VEGF-D overexpression promotes the formation of lymphatic vessels in heterotopic bone. During development, LECs are reported to arise from endothelial and nonendothelial sources (27–31). However, the origins of LECs in heterotopic bone remain unclear. LECs in heterotopic bone could arise from preexisting LECs in the wounded tissue. Indeed, it has been reported that ectopic lymphatic vessels in bone arise from preexisting LECs outside of bone in an animal model of Gorham-Stout disease (26). Unfortunately, our current mouse model uses the Cre-lox system to drive VEGF-D expression, limiting our ability to perform lineage tracing studies or conditionally knock out genes in LECs. In the future, *Hoxa11-rtTA* mice (instead of *Hoxa11^CreERT2^* mice) could be combined with lymphatic-specific and other CreER^T2^ lines to determine whether LECs in heterotopic bone arise from preexisting LECs and test whether VEGF-D activation of VEGFR3 on LECs is required for heterotopic bone resorption in our model.

There is growing interest in investigating the function of lymphatics in the pathogenesis of HO. We previously reported that lymphangiogenesis occurs during HO progression and that *Vegfc*-expressing MPCs differentiate into heterotopic bone (14). Another recent study demonstrated that lymphatics contribute to the initiation and progression of HO in an Achilles tendon tenotomy model (24). They found that a subset of Col2^+^ MPCs adopt an LEC fate during HO formation and that conditional deletion of *Fgfr3* in the Col2^+^ lineage or in LECs reduced lymphangiogenesis and increased HO formation (24). Our current work examines a distinct and previously unexplored aspect of lymphatics and HO, the role of lymphangiogenesis in promoting the resorption of established HO. We found that VEGF-D overexpression or slow release by a hydrogel increased lymphangiogenesis and HO resorption. Additionally, we found that *Hoxa11*-expressing MPCs do not differentiate into LECs in our B/T model. Taken together, the work by Zhang et al. (24) suggests that lymphatics play an important role in limiting HO formation (early stage), and our current work demonstrates that lymphangiogenesis can facilitate HO clearance (late stage).

We found that VEGF-D-induced lymphangiogenesis is associated with osteoclast-mediated bone resorption. The mechanisms by which lymphatic vessel growth triggers osteoclast-mediated bone resorption remain unclear. Prior in vitro experiments with LECs demonstrated that they express *Csf1* (m-CSF), which promotes osteoclastogenesis (32). However, we did not observe high *Csf1* expression by LECs in our scRNA-seq dataset or in three publicly available LEC scRNA-seq datasets (33–35). Moreover, we did not observe *Tnfsf11* (RANKL) expression by LECs, and *Flt4* (VEGFR3) expression was limited to LECs and blood endothelial cells. Furthermore, recombinant VEGF-D did not enhance osteoclast formation in vitro. These data suggest that LECs and VEGF-D do not directly induce osteoclast formation in our model. Lymphatic vessels may indirectly modulate osteoclast activity by altering the local cytokine milieu or by affecting immune cell trafficking. Interestingly, our scRNA-Seq data showed *Tnfsf11* expression by T cells. T cells contribute to the pathology of lymphatic diseases such as lymphedema and promote osteoclast development (36, 37). In the future, depletion experiments could determine whether T cells contribute to VEGF-D-induced osteoclast formation and bone resorption.

We found that VEGF-D induces native and heterotopic bone resorption when expressed early, but preferentially induces heterotopic bone resorption when expressed late following injury. This differential bone response may be due to differences in inflammation between early- and late-induction mice. At the time of injury, the entire limb, including native bone, tendon, fascia, and surrounding soft tissue, is exposed to a robust inflammatory response (38, 39). Inflammatory and osteoclast-lineage cells (monocytes/macrophages) accumulate broadly throughout the injured region (40), and VEGF-D overexpression during this early phase acts on a widely inflamed microenvironment. This accumulation of cells could explain why early induction of VEGF-D reduces both normal and heterotopic bone. In contrast, when VEGF-D is expressed after HO is fully established (9 wk postinjury), the inflammatory landscape is fundamentally different. Established HO remains a persistent inflammatory niche, particularly within the tendon where bone is not physiologically supposed to exist (41). Indeed, prior studies show that mature HO retains clusters of inflammatory and osteoclast-lineage cells, chronic myeloid infiltration, and elevated cytokine activity compared to surrounding tissues (1, 38–41). Native bone at this time point no longer displays significant inflammation. This difference in inflammation and presence of osteoclast-lineage cells could explain the differential responsiveness of heterotopic versus native bone to VEGF-D after HO is established.

Our finding that VEGF-D-induced lymphangiogenesis drives site-specific heterotopic bone resorption has significant therapeutic implications for the treatment of HO. We found that recombinant VEGF-D incorporated into a slow-release hydrogel induced HO resorption, suggesting this approach has potential for treating HO. Interestingly, the VEGF-D hydrogel had a greater effect on floating HO than calcaneal-associated HO. This regional effect may be due to the hydrogel being injected adjacent to the floating HO. In addition to our approach, direct injection of recombinant VEGF-D or viral-mediated VEGF-D expression in regional tissues could potentially be used to treat local disease. Notably, viral-mediated VEGF-C expression has been pursued to treat lymphedema in animal models and in humans (42–45). Thus, this approach could possibly be repurposed for treating HO in the future. Importantly, our work provides the proof-of-concept for site-directed lymphangiogenesis to induce HO resorption. Future work could focus on optimizing conditions for different pro-lymphangiogenic strategies for treating HO (e.g., AAV-VEGF-C/C or VEGF-D-containing hydrogels).

Although our study provides proof of principle that lymphangiogenesis induces HO resorption, it has several limitations. HO occurs following several different types of injury. One limitation of our study is that we examined only the effect of site-directed lymphangiogenesis on early- and late-stage HO in our B/T HO model. In the future, we will determine whether lymphangiogenesis impairs HO caused by other stimuli (e.g., different injury or *Alk2* mutations). Another limitation of our study is that we were limited to examining the effect of lymphangiogenesis on HO in the zeugopod region. This limitation results from our genetic approach targeting VEGF-D overexpression in the zeugopod region and from our surgical model that triggers HO formation in the hind limb. In the future, we will test whether other approaches to express VEGF-D (e.g., slow-release hydrogel or AAV-mediated expression) can promote HO resorption in different body sites. Last, we did not examine ankle range of motion, gait, or muscle contractility to determine whether VEGF-D-induced HO resorption improves limb function. These functional assays will be important to perform after the VEGF-D treatment strategy has been optimized.

In conclusion, our study demonstrates that site-directed lymphangiogenesis drives the therapeutic resorption of heterotopic bone in a mouse model of trauma-induced HO. In the future, site-directed lymphangiogenesis may serve as a therapeutic strategy to suppress HO or incite the resorption of existing heterotopic bone.

## Materials and Methods

Details regarding Materials and Methods can be found in the *SI Appendix*.

### Study Approval.

The animal experiments described in this manuscript were carried out in accordance with an animal protocol approved by the Institutional Animal Care and Use Committee of UT Southwestern Medical Center.

### Statistical Analysis.

Data were analyzed using GraphPad Prism statistical analysis software (Version 9.5.1). All results are expressed as mean ± SD. The number of mice in each group is indicated in the figure legends. Unpaired Student’s *t* tests were performed to test means for significance. Data were considered significant at *P* < 0.05.

## Supplementary Material

Appendix 01 (PDF)

## Data Availability

Our scRNA-seq datasets are available from Gene Expression Omnibus (GSE247763) (46). All study data are included in the article and/or *SI Appendix*.
