# Supplementary material for: VEGF-D-induced intraosseous lymphangiogenesis drives site-specific heterotopic bone resorption
Source: Proc Natl Acad Sci U S A. 2026 May 5;123(19):e2524022123. doi: 10.1073/pnas.2524022123 (PMC13167802; doi:10.1073/pnas.2524022123)
Supplement: Supplementary file 1 — Appendix 01 (PDF) [file pnas.2524022123.sapp.pdf]

Supplementary Materials:

Materials and Methods.

Supplementary Figure 1. *Vegfd* overexpression enhances lymphatic vessel invasion and bone resorption at early and late stages following burn/tenotomy injury.

Supplementary Figure 2. Injury induces lymphangiogenesis, bone loss, and osteoclast activity in *Vegfd-OE* mice.

Supplementary Figure 3. Overexpression of GFP does not impact HO formation or bone structure.

Supplementary Figure 4. VEGF-D does not directly regulate osteoclast fusion or mesenchymal lineage commitment *in vitro*.

Supplemental Figure 5. *Vegfd* overexpression does not alter baseline lymphatic vessel density in uninjured bone.

Supplemental Table 1. Top 20 genes for each cluster in scRNA-seq data.

Supplemental Table 2. Additional bone parameters for distal tibia.

Materials and Methods.

Mouse strains and genotyping

Mice were maintained in ventilated microisolator cages and were fed a standard diet. Mice were provided igloos and nestlets as enrichment items. The *Hoxa11*^{CreERT2}, *R26*$^{LSL\text{-}rtTA}$, *TetO-Vegfd*, and *TetO-eGFP* were described previously (1-4).

Tamoxifen and doxycycline administration

To induce Cre-mediated recombination, we fed mice tamoxifen-citrate chow (Teklad, TD.130860) for one week. After placing the mice on tamoxifen-citrate chow, we injected them (i.p.) with 100 µl of a tamoxifen solution on days 0 and 2. To make the tamoxifen solution, we dissolved tamoxifen (20 mg; MilliporeSigma, T5648) in a mixture of ethanol (100 µl; MilliporeSigma, E7023) and sunflower oil (900 µl; MilliporeSigma, W530285). Mice received water containing doxycycline (1 mg/ml; Sigma-Aldrich, D9891, St. Louis, MO) and sucrose (5% w/v) to induce *Vegfd* or *eGFP* expression. The water was replaced two to three times a week.

Burn/Tenotomy (B/T) procedure to induce HO

Mice were anesthetized with isoflurane, and their dorsal hair was shaved. An aluminum block heated to 60°C was applied to the dorsal region for 18 seconds to achieve a partial thickness burn injury (over 30% of the total body surface area). Each mouse also received an Achilles tendon transection at the midpoint, and a single 5-0 Vicryl suture was used to close the skin. Pain management was achieved with a subcutaneous injection of Buprenorphine Sustained-Release.

Tissue processing and immunofluorescence staining

The right and left hindlimbs from mice were collected at designated timepoints. Legs were fixed in 4% paraformaldehyde (PFA) for 24 hours at 4°C, washed with phosphate-buffered saline (PBS, Gibco, Waltham, MA), followed by immersion in 1X phosphate-buffered saline for µCT scanning. After scanning, hindlimbs were decalcified for 6 weeks with 14% EDTA (Ethylenediaminetetraacetic acid, pH 7.4) (Sigma Aldrich, St. Louis, MO). Tissues were then embedded in gelatin (20 % sucrose + 2% polyvinylpyrrolidone + 8% gelatin from porcine skin, Sigma Aldrich, St. Louis, MO), or paraffin to prepare longitudinal sections. Sections were cut at 10- or 30-microns for immunofluorescence, TRAP, and hematoxylin and eosin staining. For immunofluorescence staining, frozen sections were thawed and washed in 1X tris-buffered saline with tween-20 (TBS-T: 1xTBS (Bio-Rad, Hercules, CA), 0.05% tween-20 (Sigma Aldrich, St. Louis, MO)). Sections were blocked with donkey serum blocking solution (1% BSA, 2% goat serum, 0.1% cold water fish skin gelatin, 0.05% TritonX-100, 0.05% Tween-20, 300mM glycine, 1x TBS, pH 8.4) for 2 hours at room temperature (RT), and then incubated at 4°C overnight with an antibody against LYVE1 (R&D Systems; AF2125, 1:100). Slides were washed in 1X TBS-T 3 times and incubated with fluorescence-conjugated secondary antibodies for 2 hours at RT (Invitrogen donkey anti-goat AF488; 1:500). Then, slides were mounted with ProLong® Gold Antifade Reagent with DAPI and #1.5 Slip-Rite cover glass. Appropriate primary antibody and negative controls were run simultaneously with each tested sample. For immunofluorescence imaging of 10-micron murine sections, 1x zoom images were captured with a 10x and 40x oil-immersion lens on a Leica SP8 confocal microscope using Leica's LAS X software.

Confocal microscopy and image analysis

Confocal microscope images were acquired with a Leica Stellaris DMI8 microscope equipped with the HC PL APO CS2 10x or 40x objectives. The raw channel images, including DAPI and the specific antigens of interest, were exported using the Leica Application Suite software. For lymphatic quantification, each raw image from selected ROI was processed as an 8-bit image

within ImageJ software. Percentage area measurements were performed using consistent manual thresholding parameters across all experiments.

Tartrate-resistant acid phosphatase (TRAP) staining

Samples were submitted to the Histology Core at UT Southwestern Medical Center for TRAP staining. TRAP-stained slides were scanned using Hamamatsu NanoZoomer 2.0-HT (Hamamatsu, Hamamatsu City, Shizuoka, Japan). For quantification, TRAP-positive multinucleated osteoclasts (defined as cells with ≥3 nuclei) on the bone surface within HO or native bone were manually counted. The bone surface length was measured using NDP.view2 image analysis software. Osteoclast numbers were normalized to bone surface area and expressed as the number of osteoclasts per millimeter of bone surface (No. Oc/mm). Quantification was performed on at least three non-consecutive sections per sample by an observer blinded to experimental conditions.

Osteoclast Differentiation and TRAP Staining

Primary bone marrow–derived monocytes/macrophages were isolated from femurs and tibiae of C57BL/6 mice by flushing bone marrow cavities with sterile PBS using a 27-gauge needle. Cells were passed through a 70-µm cell strainer, centrifuged, and resuspended in complete α-MEM containing 10% FBS, 2 mM glutamine, and 1% penicillin/streptomycin. Cells were initially plated in complete medium supplemented with 2 ng/ml recombinant mouse macrophage colony-stimulating factor (M-CSF; R&D Systems) to enrich for monocytes/macrophages. After 24 hours, non-adherent cells were collected, centrifuged, and seeded at a density of 1×10^6 cells per well in 12-well tissue culture plates in complete medium supplemented with 20 ng/ml M-CSF and 10 ng/ml recombinant mouse RANKL (R&D Systems) to induce osteoclast differentiation. Where indicated, recombinant mouse VEGF-D (100 ng/ml) was added to the differentiation medium. Cultures were maintained at 37°C with 5% CO_2, and medium was refreshed every 2–3 days. At days 8–10, cells were fixed and stained using a tartrate-resistant acid phosphatase (TRAP) staining kit (Sigma-Aldrich, #387A) according to the manufacturer's instructions. Following TRAP staining, a hematoxylin counterstain was performed to visualize nuclei. TRAP-positive multinucleated cells containing three or more nuclei were identified as mature osteoclasts. Quantification was performed by manual counting of osteoclasts from stained microscopic images, and data are presented as the number of $TRAP^+$ multinucleated osteoclasts per well.

Osteoblast Differentiation and Mineralization Assays

Osteoprogenitor cells were cultured in osteogenic differentiation medium (ODM) to induce osteoblast differentiation. Osteogenic medium consisted of basal culture medium supplemented with standard osteogenic additives and was refreshed every 2–3 days. Where indicated, recombinant mouse VEGF-D was added to the differentiation medium at final concentrations of 1, 10, or 100 ng/ml. Cultures were maintained at 37°C with 5% CO_2 throughout the differentiation period.

Alkaline Phosphatase (ALP) Staining and Activity

For assessment of early osteogenic differentiation, cells were washed once with 1× PBS and fixed with 10% neutral buffered formalin for 20–60 minutes at room temperature. Following fixation, cells were washed with distilled water and incubated with freshly prepared alkaline phosphatase (ALP) staining solution protected from light. The ALP dye solution was prepared according to standard protocols using sodium nitrate solution, FRV-alkaline solution, and naphthol AS-BI alkaline solution, mixed immediately before use. Cells were incubated in ALP staining solution for at least 1 hour at room temperature in the dark. Staining was terminated by washing with distilled water, and plates were air-dried prior to imaging.

ALP enzymatic activity was quantified separately and normalized to total protein content, and results are reported as units per gram of protein.

Alizarin Red S (ARS) Staining

For evaluation of late-stage osteogenic differentiation and matrix mineralization, cells were washed with 1× PBS and fixed with 10% neutral buffered formalin for 1 hour at room temperature. After fixation, cells were washed thoroughly with distilled water to remove residual fixative. Cells were then incubated with Alizarin Red S staining solution (prepared in water at the appropriate pH) and protected from light for at least 1 hour at room temperature. Following staining, excess dye was carefully removed, and cells were washed multiple times with distilled water until background staining was minimized. Plates were air-dried and stored protected from light until imaging. Quantification of Alizarin Red S staining was performed by measuring mean staining intensity across wells, and values were compared across treatment groups.

μCT analysis

μCT imaging was conducted using a Bruker SKYSCAN 1272 desktop μCT scanner, using settings of X-ray power at 70 kV and 142 μA, and a 0.5 mm Al filter. Scans were performed at a high resolution of 7.25 μm. These images were subsequently reconstructed using the Bruker NRecon Reconstruction software at a voxel size of 0.45 μm. The μCT-generated images were stored as a sequence of DICOM files, and these files were uploaded into CTAn Version 1.23 To quantify different parameter of the bone, including: HO bone volume and porosity, Dital tibia BV/TV and porosity and Calcaneus bone BV/TV and porosity.

Tissue digestion and single-cell RNA sequencing

Male and female control and *Vegfd-OE* mice underwent the B/T procedure when they were between 8-12 weeks old (n=5/group). Mice were euthanized 7 days later for tissue collection. Following euthanasia, tissue from the injury site was collected by resecting the soft tissue located posterior to the tibia between the insertion and origin of the Achilles tendon. Cells from the excised tissue were isolated based on previously described methods. Briefly, tissue was digested for 45 min in 0.3% Type 1 Collagenase and 0.4% Dispase II (Gibco) in Roswell Park Memorial Institute (RPMI) medium at 37 °C under constant agitation at 120 rpm. Digestions were quenched with 10% FBS RPMI and filtered through 40μm sterile strainers. Cell viability was assessed with Trypan blue exclusion on a Countess II (Thermo Fisher Scientific) automated counter, and samples with >85% viability were processed for further sequencing. Single-cell 3' library generation was performed on the 10X Genomics Chromium Controller following the manufacturers' protocol for the v2 reagent kit (10X Genomics, Pleasanton, CA, USA). Cell suspensions were loaded onto a Chromium Single-Cell A chip along with reverse transcription (RT) master mix and single cell 3' gel beads, aiming for 2000–6000 cells per channel. Following the generation of single-cell gel bead-in-emulsions (GEMs), reverse transcription was performed, and the resulting Post GEM-RT product was cleaned using DynaBeads MyOne Silane beads (Thermo Fisher Scientific, Waltham, MA, USA). The cDNA was amplified, SPRIselect (Beckman Coulter, Brea, CA, USA) cleaned, quantified, and then enzymatically fragmented and size selected using SPRIselect beads to optimize the cDNA amplicon size prior to library construction. An additional round of double-sided SPRI bead cleanup was performed after end repair and A-tailing. Another single-sided cleanup was done after adapter ligation. Indexes were added during PCR amplification and a final double-sided SPRI cleanup was performed. Libraries were quantified by Kapa qPCR for Illumina Adapters (Roche) and size was determined by Agilent tapestation D1000 tapes. Read 1 primer sequence were added to the molecules during GEM incubation. P5, P7 and sample index and read 2 primer sequence were added during library construction via end repair, A-tailing, adaptor ligation and PCR. Libraries were generated with unique sample indices (SI) for each sample. Libraries were sequenced on a HiSeq 4000,

(Illumina, San Diego, CA, USA) using a HiSeq 4000 PE Cluster Kit (PN PE-410-1001) with HiSeq 4000 SBS Kit (100 cycles, PN FC- 410-1002) reagents, loaded at 200 pM following Illumina's denaturing and dilution recommendations. The run configuration was 26 × 8 × 98 cycles for Read 1, Index and Read 2, respectively. Cell Ranger Single Cell Software Suite 1.3 was used to perform sample de-multiplexing, barcode processing, and single cell gene counting (Alignment, Barcoding and UMI Count) at the University of Texas Southwestern Medical Center Genomics Sequencing Core Facilities.

Single-cell RNA sequencing analysis

Cell Ranger (v5.0.1) software from 10X Genomics Inc. was used to align reads to mm10-2020-A reference genome to generate matrices for *Vegfd-OE* uninjured and post-tenotomy Day 7 Achilles tendon samples. ScRNA-seq data for C57BL/6 (WT) uninjured and post-B/T Day 7 samples were obtained from GSE126060 on NCBI GEO. Downstream analysis was performed using the Seurat (v5.0.1) R package. Cells with fewer than 500 genes or more than 25% mitochondrial read content were filtered out. Subsequent analysis included normalization, identification of highly variable genes, scaling based on UMI counts and batch effects, dimensionality reduction (PCA, UMAP), unsupervised clustering, and differential expression analysis. Harmony was used to perform integration between datasets, aligning and clustering cells from different sample types. Differentially expressed genes between aligned clusters were identified using a negative binomial test. An osteoclast differentiation score was generated using the AddModuleScore function of Seurat and calculated as the level of gene expression enrichment of a set gene list–composed of the following genes: *Mmp9*, *Acp5*, *Calcr*, *Oscar*, *Ocstamp*, *Dcstamp*, *Ctsk*, *Atp6ap1*, *Tnfrsf11a*, *Tnfrsf11b*, *Itgb3*, *Csf1r*, *Csf1*, *Tcirg1*, *Siglec15*, *Trem2*, *Tyrobp*, *Atp6v0d2*–relative to a random control list within monocytes, macrophages, OC precursors, and osteoclasts, with higher module score values representing positive enrichment beyond background, and compared between control and *Vegfd-OE* mice. Pseudotime analysis of myeloid cells, pre-OCs, and OCs was performed using Monocle3 to identify the differentiation trajectory within myeloid cells, with semi-automated root cell identification. To assess MPC differentiation dynamics, MPCs were subset based on canonical mesenchymal marker expression and subjected to trajectory inference using the Palantir algorithm. Palantir models differentiation as a stochastic process in which progenitor cells transition toward terminally differentiated states through intermediate phenotypic stages on a low-dimensional manifold. Trajectory directionality and lineage commitment toward tenocyte, osteoblast, and chondrocyte fates were assessed independently for WT and *Vegfd-OE* samples. To further quantify lineage bias, the Lamian pipeline was applied to infer pseudotime tree structure and calculate branch detection rates (trajectory probabilities) for each differentiation direction, which were compared between WT and *Vegfd-OE* groups.

Data and materials availability

Our scRNA-seq datasets are available from Gene Expression Omnibus (GSE247763).

References

1. K. M. Pineault, J. Y. Song, K. M. Kozloff, D. Lucas, D. M. Wellik, Hox11 expressing regional skeletal stem cells are progenitors for osteoblasts, chondrocytes and adipocytes throughout life. *Nat Commun* **10**, 3168 (2019).
2. G. M. Lammoglia *et al.*, Hyperplasia, de novo lymphangiogenesis, and lymphatic regression in mice with tissue-specific, inducible overexpression of murine VEGF-D. *American journal of physiology. Heart and circulatory physiology* **311**, H384-394 (2016).
3. L. E. Dow *et al.*, Conditional reverse tet-transactivator mouse strains for the efficient induction of TRE-regulated transgenes in mice. *PLoS One* **9**, e95236 (2014).

4. T. Tumbar *et al.*, Defining the epithelial stem cell niche in skin. *Science* **303**, 359-363 (2004).

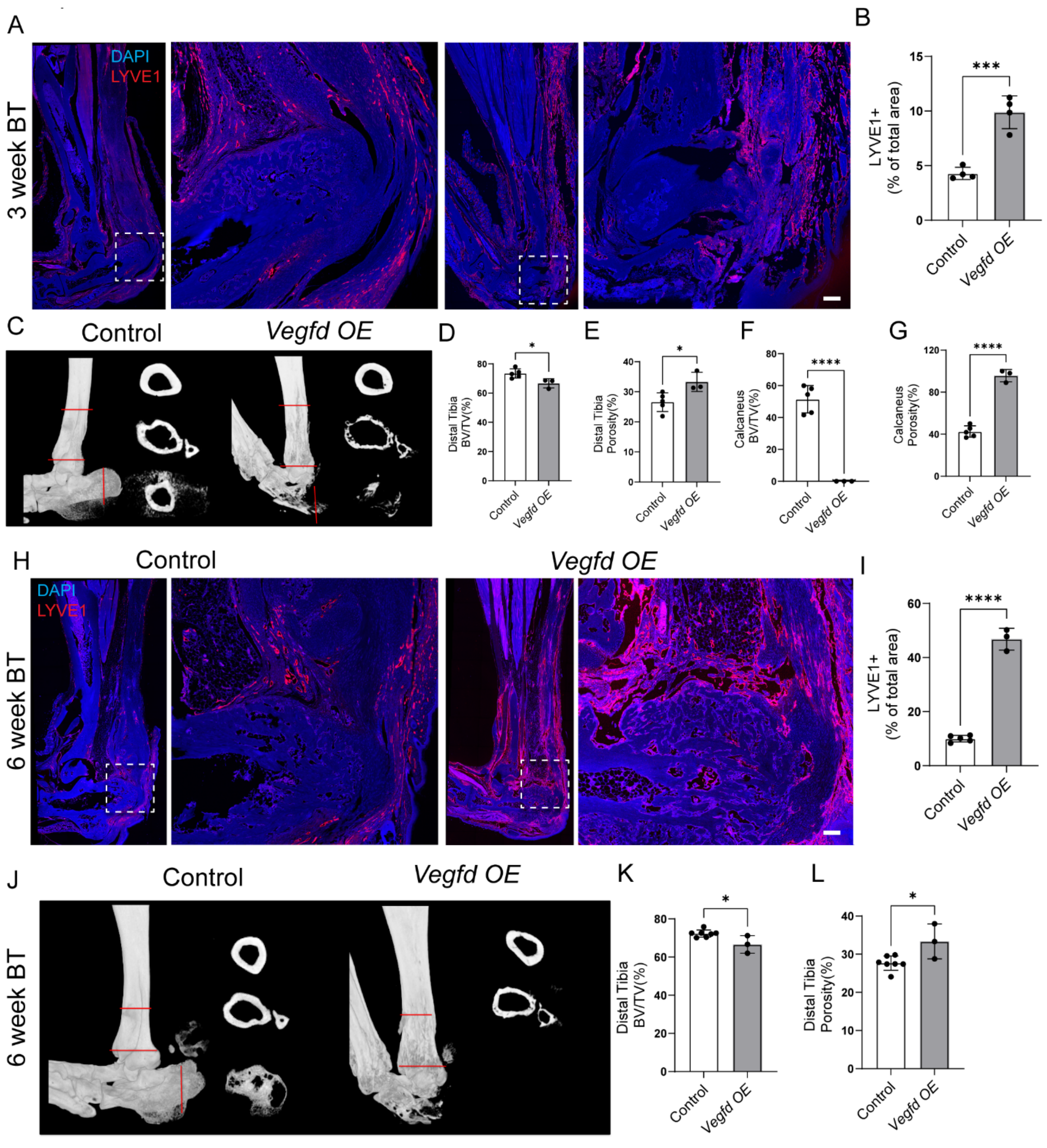

Supplementary Figure 1: *Vegfd* overexpression enhances lymphatic vessel invasion and bone resorption at early and late stages following burn/tenotomy injury. (A) Representative immunofluorescence images of Achilles tendon HO lesions at 3 weeks B/T in control and *Vegfd-OE* mice stained for DAPI (blue) and LYVE1 (red). Dashed boxes indicate regions shown at higher magnification. **(B)** Quantification of LYVE1^{+} area normalized to total tissue area at 3 weeks B/T. **(C)** Representative µCT reconstructions of hindlimbs from control and *Vegfd-OE* mice at 3 weeks B/T. Red lines indicate region of interest used for quantification. **(D-G)** Quantification of distal tibial BV/TV and porosity and calcaneal BV/TV and porosity respectively. **(H)** Representative immunofluorescence images of HO lesions at 6 weeks B/T stained for DAPI and LYVE1. **(I)** Quantification of LYVE1^{+} area normalized to total tissue area at 6 weeks B/T. **(J)** Representative µCT reconstructions of hindlimbs at 6 weeks B/T. **(K-L)** Quantification of distal and proximal tibial BV/TV.

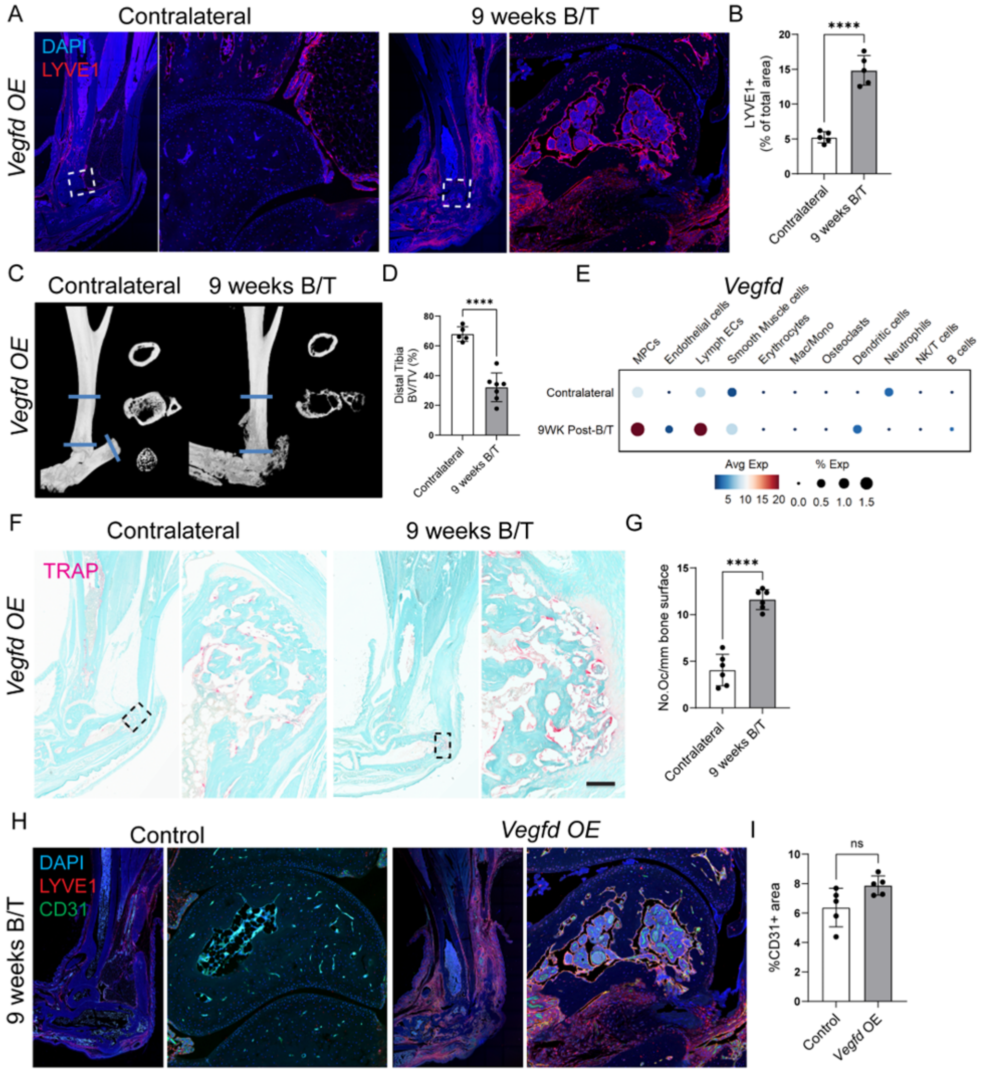

Supplementary Figure 2. Injury induces lymphangiogenesis, bone loss, and osteoclast activity in *Vegfd-OE* mice. (**A**) Representative immunofluorescence images of contralateral (uninjured) and 9-week post-B/T limbs from *Vegfd OE* mice. Sections are stained for LYVE1 (red), and DAPI (blue). Insets show higher magnification of lymphatic vessel regions. (**B**) Quantification of LYVE1^{+} area as a percentage of total tissue area. (**C**) Representative μCT images of contralateral and 9-week B/T-injured limbs from *Vegfd-OE* mice. Blue lines indicate the regions for cross-section images. (**D**) Quantification of distal tibia bone BV/TV shows significant bone loss following injury. (**E**) Dot plot showing Vegfd expression in scRNA-seq data sets for injured and uninjured (contralateral) limbs from *Vegfd-OE* mice. (**F**) Representative TRAP-stained histological sections showing osteoclast presence on bone surfaces. More TRAP^{+} osteoclasts (red) are visible in injured limbs compared to contralateral controls. (**G**) Quantification of osteoclast number (No. Oc/mm bone surface) confirms significantly increased osteoclast activity post-injury. Data are shown as mean ± SD. Unpaired Student's t-test. $p < 0.0001$ (****). Each point on the graph corresponds a data point collected from a single mouse (n = 5–7 mice/group). **(H)** Representative immunofluorescence images of control and *Vegfd-OE* limbs stained for DAPI, LYVE1, and CD31 at 9 weeks post-B/T. **(I)** Quantification of CD31^{+} area normalized to total tissue area.

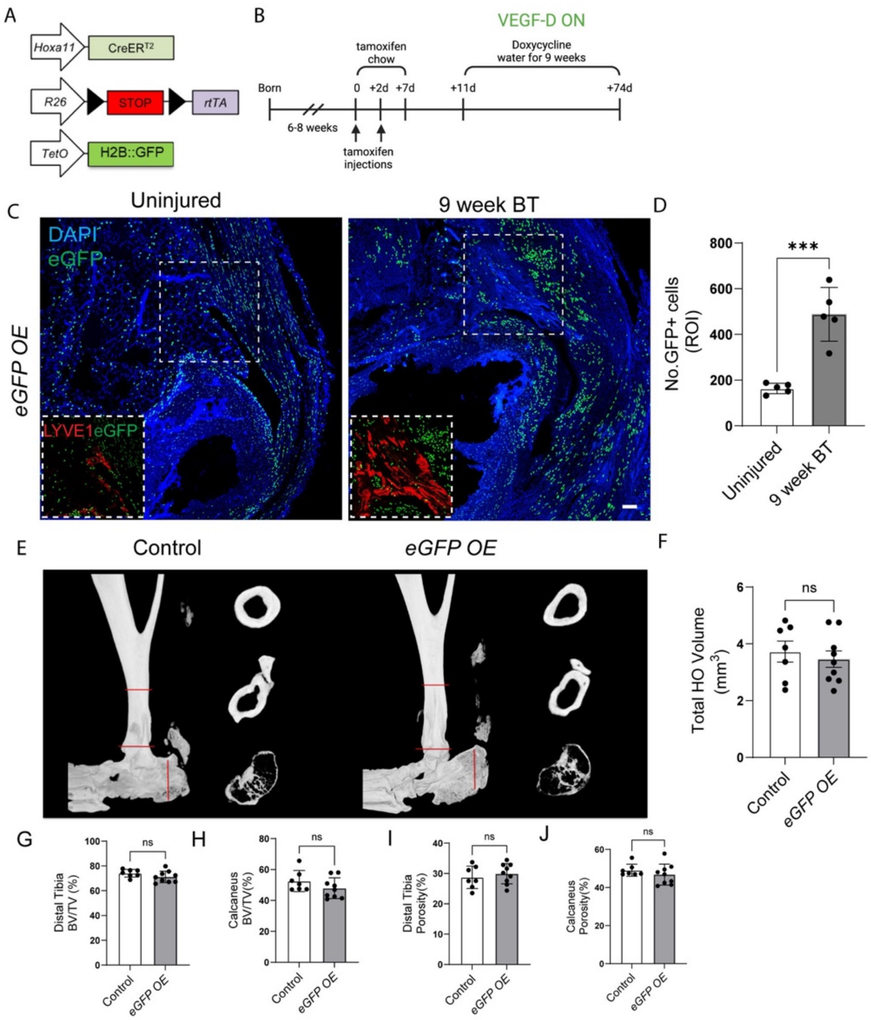

Supplementary Figure 3. Overexpression of GFP does not impact HO formation or bone structure. (**A**) Schematic of transgenic mouse model for doxycycline-inducible eGFP expression using *Hoxa11-CreER^{T2}; Rosa26-rtTA; TetO-eGFP* alleles. (**B**) Experimental timeline: tamoxifen was administered to adult mice, followed by 9 weeks of doxycycline to induce H2B::eGFP expression. (**C**) Immunofluorescence images of LYVE1 (red), eGFP (green), and DAPI (blue) in uninjured and 9-week post-BT tissues. Inset shows *eGFP*-expressing cells adjacent to lymphatic vessels. (**D)** Quantification of eGFP+ cells in the region of interest (ROI) shows significant increase in 9-week post-BT samples compared to uninjured controls. (**E**) Representative µCT images of hindlimbs from control and *eGFP* OE mice at 9 weeks post-BT. (**F-J**) Quantification of HO volume (**F**), bone volume/tissue volume (BV/TV) in distal tibia (**G**) and calcaneus (**H**), and porosity in distal tibia (**I**) and calcaneus (**J**) shows no significant differences between control and *eGFP* OE groups. Data are presented as mean ± SD. Unpaired Student's t-test. ns = not significant, $p < 0.001$ (***). Each point on the graph corresponds a data point collected from a single mouse (n = 5–9 mice/group).

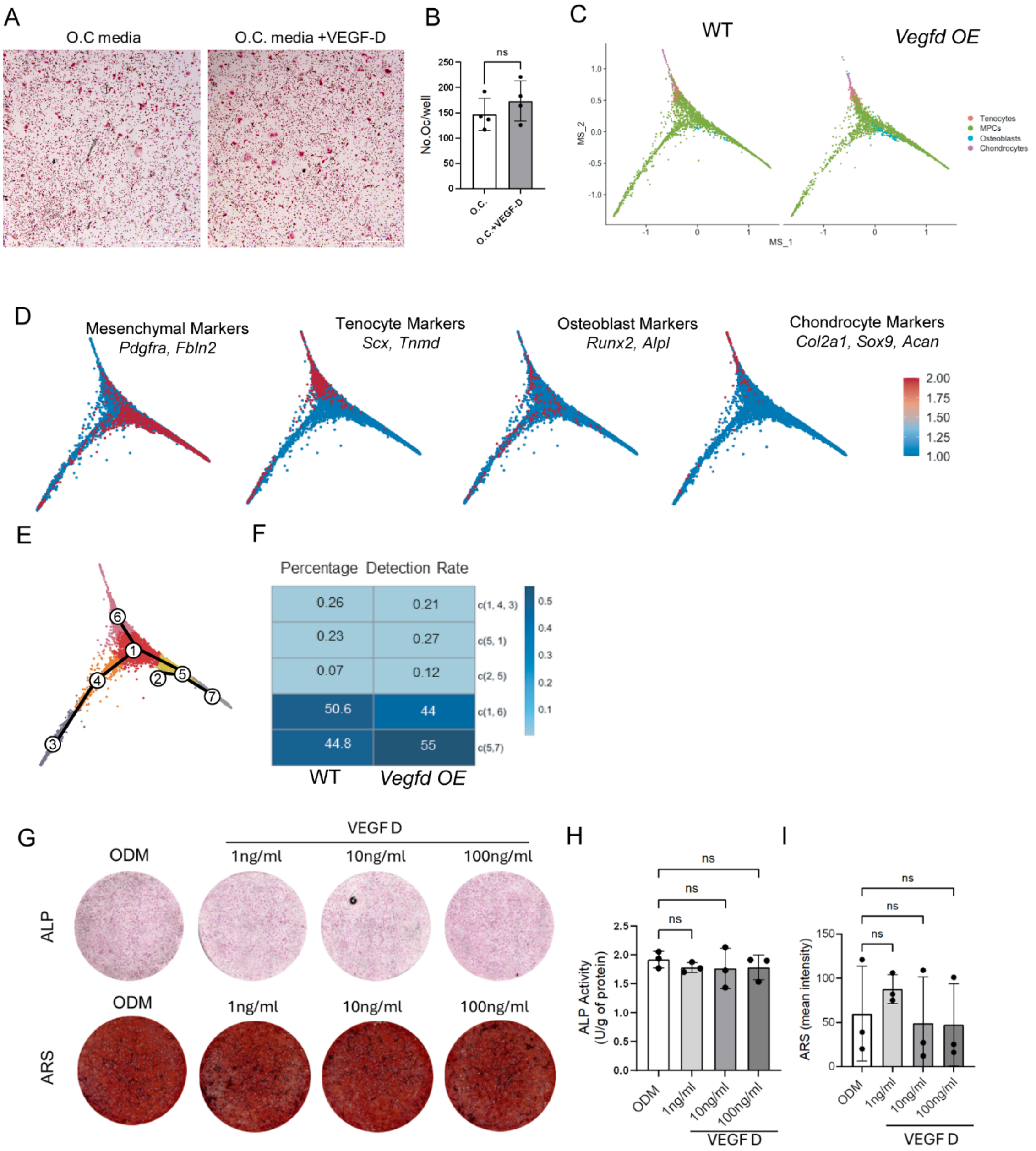

Supplementary Figure 4. VEGF-D does not directly regulate osteoclast fusion or mesenchymal lineage commitment *in vitro*. (A) Representative TRAP-stained images of osteoclast cultures treated with osteoclast media alone or osteoclast media supplemented with VEGF-D. **(B)** Quantification of TRAP$^+$ multinucleated osteoclasts per well. **(C)** UMAP visualization of MPCs from WT and *Vegfd-OE* samples. **(D)** Feature plots showing expression of mesenchymal, tenocyte, osteoblast, and chondrocyte marker genes. **(E)** Pseudotime trajectory of MPC differentiation. **(F)** Heatmap showing percentage detection rate of differentiation trajectories toward indicated lineages in WT and *Vegfd-OE* groups. **(G)** Representative ALP and ARS staining of osteogenic cultures treated with increasing concentrations of VEGF-D. **(H)** Quantification of ALP activity. **(I)** Quantification of ARS mean intensity. One-way ANOVA. ns = not significant. Each point on the graph corresponds a data point collected from a single well (n = 3 wells/group).

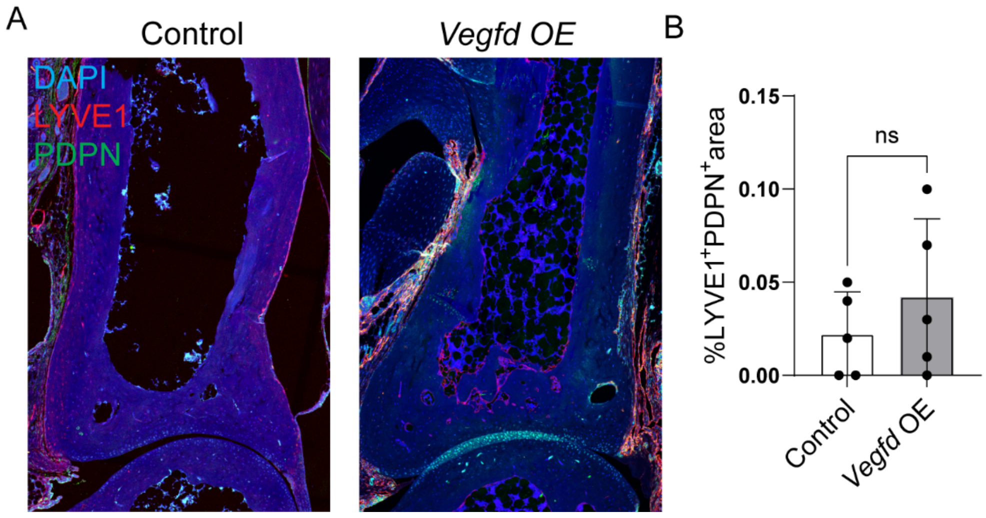

Supplemental Figure 5. *Vegfd* overexpression does not alter baseline lymphatic vessel density in uninjured bone. (A) Representative immunofluorescence images of control and *Vegfd-OE* tibia bone stained for DAPI, LYVE1, and PDPN. **(B)** Quantification of LYVE1$^+$PDPN$^+$ area normalized to total tissue area.

Table 1. Top 20 genes for each cluster in scRNA-seq data.

T cells	Foxp3- Icos- Klrg1- Cd3g- Cd3e -Themis- Cd6 -Pdcd1 -Cxcr6 -Cd3d -Trbc2 -Cd5 -Tnfrsf4<br>Trac -Ikzf3 -Sit1 -Lat -Il2ra -Ctla4 -Itk
NK cells	Klra7- Klra4 - Klra8 - Klra9 – Gzma -Ncr1 -Xcl1 -Prf1 -Klrb1c -Eomes -Cd160 -Klrc2 -Samd3 -Klrc1 -Klre1 -Nkg7 -Ctsw -Gimap7 -Klrb1f -Ifng
Tenocytes	Kera- Chst5 – Lipk - Gm17455 -Fmod -Cybrd1 -Tnmd -Scx -Mkx -Thbs4 -Comp -Scube2<br>Zfp185 – Ucma - Galnt5 -Nudt11 -Isl1 -Ecrg4 -C1qtnf3 -Kcnk1
Macrophages	Ccl8 -Cbr2 -Folr2 -Ccl12 -Fcrls -Npl -Hpgds -Syngr1 -C1qc -Mrc1 -Apoe -Rapsn -F13a1<br>Arhgap19 -Gpr34 -Aoah -Pf4 -C1qa -Tmem8 -Ms4a7
Endothelial cells	Sele- Tacr1- Gm12002- Tmem252 -Gpihbp1 -Adgrl4 -Sox17- Depp1- Cyyr1- Lhx6 -Dipk2b – Aplnr- AU021092 -Nr5a2 -Rbp7 – Ptprb - Fam167b - Grrp1 - Syt15 - Robo4
MPCs	Clec3b- Sfrp4 -Dpep1 -Sfrp2 - Mmp3 – Cpz - Scara5 - Cxcl5- Pi16- Col14a1- Tmeff2 -Dpt- AW551984- Slc1a3- Tnxb- Osr1- Pcolce2- Serpina3n- Has1- Sfrp1
Keratinocytes	Pla2g2f- Skint1- Sdr16c6- Dsg1b- Abca12- Krt1- H60c- Skint4- Dsg1a-4833423E24Rik<br>Dsg3- Csta1- Krt15- Calm4- Tgm5- Fam25c- Fam83b- Calml3- Krt6a- Prom2
Osteoclasts	Cyp2s1- Slc9b2- Acp5- Mmp9- Hist1h1b- Ocstamp- Atp6v0d2- Esco2- Hist1h3c<br>Pclaf- Chchd10- Sgo1- Hist1h2ae- Abcb4- Nusap1- Dnph1- Knl1- Top2a- Brca1-Kif11
Neutrophils	Cstdc4- Stfa2l1- Mirt2- Acod1- Cxcr2- Retnlg- 4933432I03Rik- Spatc1-4732465J04Rik<br>S100a9- 5430425K12Rik- Nlrp12- Rdh12- Il1f9- S100a8- Fpr1- Cxcr5- Mirt1- G0s2-Dhrs9
OC Precursors	Mmp12- Arg1- F7- Rnf128- Spp1- Kcnn4- F10- Itgax- Lgals3- Gpnmb- AA467197-Msr1<br>Ccl9- Cd36- Lpl- Il1rn- Adam8- Clec5a- Prdx1 -Cstb
Monocytes	Gm9733- H2-Ab1- H2-Eb1- H2-Aa- Napsa- Cd74- Ms4a4c- H2-DMb1- Ccr2- Plbd1<br>Ifitm6- H2-DMa- Tnip3- Clec4b1- Lsr- Bcl2a1d- Sirpb1c -Ly6c2- Plac8 -Gpr141
Lymph ECs	Ccl21a- Mmrn1- Prox1- Dtx1 -Scn5a -Apba2 -Tc2n -Ackr2 -Reln -Flt4 -Scn3a -Slc45a3<br>Sh3gl3 -Susd4 -Lyve1 -Tbx1 -Gpm6a -Klhl4 -Fndc8 -Il7
Dendritic cells	Dntt- D13Ertd608e- Siglech -Klk1b27 -Ccr9 -Pacsin1 -Cox6a2 -Mzb1 -Cd8b1 -Cd300c -Klk1 -Cd8a -Havcr1 -Spib -Paqr5 -Smim5 -Upb1 -Sla2 - Atp2a1 - Fcrla
Smooth Muscle cells	Myh11- Olfr558- Higd1b- Tusc5- Kcnk3- Lmod1- Trarg1- Rgs5- Cox4i2 -Gucy1a3 -Gucy1b3- Gm13889 -Slc2a4 -Nrip2 -Map3k7cl -Notch3 -Trpc6 -Gucy1a1 -Gucy1b1 Atp1b2
Nerve cells	Foxd3- Gpr37l1- Kcna1- Mpz- Gm12688- Gjc3- Ugt8a- Mal- Crym- Cmtm5- Sox10-Fgf5<br>Cldn19- Cdh19- Zfp536- Plp1- Hcn1- Nkain2- Igsf11- Mlip
Osteoblasts	Wif1- Frem1- Robo2- Prg4- Rspo3- Enpp2- Prr16- Ptch1- Galnt17- Thrb- Rspo2-Kif26b<br>Mmp16- Lama2- Col23a1- Aff2- Fhod3- Kcnma1- 2610307P16Rik- Naaladl2
Chondrocytes	Col2a1- Col9a3- Ucma- Col11a2- Hapln1- Matn3- Col9a1- Frzb- Lipk- Scrg1-Wfdc18<br>Col9a2- Sbspon- Matn4- Comp- Rbp4- Kcns1 -Acan -C1qtnf3 -Moxd1
B cells	Pax5-Tpsb2- Mrgprb2- Mrgprb1- Mrgprx2- Cma1- Cpa3- Mcpt4- Fcer1a- Fcmr-Ms4a2<br>Igkc- Ighd- Ms4a1- Il4- Cd200r3- Pou2af1- Iglc2- Slc6a4- Cd79a

Table 2. Additional bone parameters for distal tibia (green font indicate mutant values).

Animal ID	Pixel size um	Percent bone volume	Total porosity (percent)	Standard deviation of trabecular thickness	Standard deviation of trabecular separation	Moment of inertia (x)	Moment of inertia (y)	Moment of inertia (z)	Polar moment of inertia	Radius of gyration (x)
		BV/TV(%)	Po(tot)(%)	SD(Tb.Th)	SD(Tb.Sp)	MMI(x)	MMI(y)	MMI(z)	MMI(polar)	Gr.R(x)
3 weeks										
Control-1664	7.25010694	69.99438129	30.00561871	0.03807039	0.09098502	0.04677735	0.06018151	0.07182437	0.08939162	0.3462299
Control-1665	7.25010694	73.10824895	26.89175105	0.04684344	0.09457798	0.06956506	0.05421211	0.08389494	0.10383606	0.39611403
Control-1666	7.25010694	71.55780192	28.44219808	0.0377706	0.09198249	0.0638185	0.04751146	0.07436534	0.09284765	0.39350443
Control-1667	7.25010694	78.11759977	21.88240023	0.04135903	0.09611891	0.09739183	0.06517715	0.1138683	0.13821864	0.42390132
Control-1694	7.25010694	74.4232063	25.5767937	0.04022639	0.09263694	0.06300486	0.06127088	0.08315992	0.10371783	0.36926073
Mutant-1695	7.25010694	68.69729994	31.30270006	0.04267392	0.14029373	0.14109666	0.10968955	0.19330344	0.22204483	0.46930835
Mutant-1907	7.25010694	68.36241106	31.63758894	0.04624292	0.11156744	0.12743801	0.0681769	0.14691553	0.17126522	0.48215619
Mutant-1927	7.25010694	62.98025356	37.01974644	0.03721431	0.13454037	0.10076421	0.07617477	0.1313653	0.15415214	0.44664946
6 weeks										
Control-1693	7.25010694	70.19736821	29.80263179	0.03534687	0.0907746	0.06113316	0.05816925	0.08138757	0.10034499	0.38065052
Control-1696	7.25010694	72.53800005	27.46199995	0.03648971	0.09266571	0.06457893	0.05194739	0.07769347	0.09710989	0.38510286
Control-1767	7.25010694	72.58988549	27.41011451	0.04117594	0.11168293	0.07568467	0.05664925	0.09067425	0.11150408	0.40310795
Control-1768	7.25010694	70.43724468	29.56275532	0.03173318	0.10068127	0.05450586	0.06449976	0.08133003	0.10016782	0.36050125
Control-1769	7.25010694	72.59160757	27.40839243	0.03630768	0.10017209	0.06057743	0.05894606	0.08068512	0.10010431	0.37376217
Control-1770	7.25010694	72.13208442	27.86791558	0.04073112	0.1014032	0.08807196	0.06653334	0.11021033	0.13240782	0.42216929
Control-1771	7.25010694	75.90383703	24.09616297	0.03844246	0.09894812	0.07952654	0.07236273	0.10634464	0.12911695	0.3946013
Mutant-1772	7.25010694	66.66789732	33.33210268	0.03643229	0.12070084	0.09184774	0.08456208	0.13009631	0.15325306	0.42321156
Mutant-1811	7.25010694	71.20411058	28.79588942	0.06524686	0.14073789	0.10772923	0.06316719	0.1242055	0.14755096	0.45576977
Mutant-1903	7.25010694	62.03116951	37.96883049	0.04501486	0.14462239	0.06556866	0.07964882	0.10489926	0.12505837	0.38139093
9 weeks	Pixel size	Percent BV/TV(%)	Total porosity %	Trabecular thickness Tb.Th	Trabecular separation Tb.Sp	Trabecular number Tb.N	Intersection surface	Bone surface / volume ratio	Bone surface density	

Control-1815	7.500075	75.85803502	35.85803502	0.08206614	0.10610053	5.70498917	6.02757687	55.49967008	25.98419179	
Control-1816	7.500075	68.26566963	28.26566963	0.0936413	0.06524765	6.36223905	7.70609728	45.01796243	26.8202771	
Control-1576	7.500075	74.25296506	24.25296506	0.12399763	0.06046886	6.00439788	9.18212187	30.03818467	19.28157744	
Control-1646	7.500075	74.35109518	34.35109518	0.09788187	0.07064078	6.22069044	13.81976123	32.45746247	21.65586717	
Control-1649	7.500075	64.79437034	31.79437034	0.12289863	0.07897205	5.76565675	7.25358623	43.44660952	24.51923567	
Mutant-1929	7.500075	35.85803502	65.85803502	0.12595525	0.11006201	5.27984691	2.23151781	26.12919569	19.97612618	
Mutant-1577	7.500075	48.26566963	48.26566963	0.13349215	0.08508222	4.99724927	3.22132739	25.42092504	16.95813471	
Mutant-1578	7.500075	34.25296506	54.25296506	0.11995731	0.17517111	5.70347177	4.7388865	28.13609761	22.20652423	
Mutant-1579	7.500075	34.35109518	44.35109518	0.13536722	0.07782427	5.52652456	6.40904857	23.91039308	21.04890333	
Mutant-1645	7.500075	24.79437034	54.79437034	0.1454539	0.09326804	3.56777721	4.25222315	22.07518417	20.16714351	
Mutant-1647	7.500075	29.35109518	39.35109518	0.15559053	0.17668285	4.36965522	4.20472234	27.59296648	15.00840965	
Mutant-1648	7.500075	17.79437034	37.79437034	0.12871166	0.09257596	5.54670587	2.23151781	28.0752474	19.69932806	
